# Chest X-ray Features of HIV-Associated *Pneumocystis* Pneumonia (PCP) in Adults: A Systematic Review and Meta-analysis

**DOI:** 10.1093/ofid/ofae146

**Published:** 2024-03-18

**Authors:** Nicola K Wills, Marguerite Adriaanse, Shandri Erasmus, Sean Wasserman

**Affiliations:** Department of Medicine, University of Cape Town, Cape Town, South Africa; Department of Medicine, University of Cape Town, Cape Town, South Africa; Victoria Hospital Wynberg, Cape Town, South Africa; Infection and Immunity Research Institute, St George's University of London, London, UK; Centre for Infectious Diseases Research in Africa, Institute of Infectious Disease and Molecular Medicine, University of Cape Town, Cape Town, South Africa; MRC Centre for Medical Mycology, Faculty of Health and Life Sciences, University of Exeter, Exeter, UK

**Keywords:** chest x-ray, HIV, PCP, *Pneumocystis jirovecii*, radiology

## Abstract

**Background:**

The performance of chest x-ray (CXR) features for *Pneumocystis* pneumonia (PCP) diagnosis has been evaluated in small studies. We conducted a systematic review and meta-analysis to describe CXR changes in adults with HIV-associated laboratory-confirmed PCP, comparing these with non-PCP respiratory disease.

**Methods:**

We searched databases for studies reporting CXR changes in people >15 years old with HIV and laboratory-confirmed PCP and those with non-PCP respiratory disease. CXR features were grouped using consensus terms. Proportions were pooled and odds ratios (ORs) generated using random-effects meta-analysis, with subgroup analyses by CD4 count, study period, radiology review method, and study region.

**Results:**

Fifty-one studies (with 1821 PCP and 1052 non-PCP cases) were included. Interstitial infiltrate (59%; 95% CI, 52%–66%; 36 studies, n = 1380; *I*^2^ = 85%) and ground-glass opacification (48%; 95% CI, 15%–83%; 4 studies, n = 57; *I*^2^ = 86%) were common in PCP. Cystic lesions, central lymphadenopathy, and pneumothorax were infrequent. Pleural effusion was rare in PCP (0%; 95% CI, 0%–2%). Interstitial infiltrate (OR, 2.3; 95% CI, 1.4–3.9; *I*^2^ = 60%), interstitial–alveolar infiltrate (OR, 10.2; 95% CI, 3.2–32.4; *I*^2^ = 0%), and diffuse CXR changes (OR, 7.3; 95% CI, 2.7–20.2; *I*^2^ = 87%) were associated with PCP diagnosis. There was loss of association with alveolar infiltrate in African studies.

**Conclusions:**

Diffuse CXR changes and interstitial–alveolar infiltrates indicate a higher likelihood of PCP. Pleural effusion, lymphadenopathy, and focal alveolar infiltrates suggest alternative causes. These findings could be incorporated into clinical algorithms to improve diagnosis of HIV-associated PCP.

*Pneumocystis* pneumonia (PCP) is a severe HIV-associated opportunistic infection caused by the ubiquitous fungus *Pneumocystis jirovecii*. Globally, PCP accounts for 5%–30% [1–3] of respiratory admissions in adults with HIV and carries an estimated case fatality rate of 10%–31% [1, 4–7], increasing up to 62% in adults requiring intensive care [5, 8]. This wide mortality range may reflect heterogeneous disease phenotype, varying clinical settings and care, and frequent respiratory coinfections, described in up to 25% of adults with PCP [1, 4]. Lack of access to bronchoscopy and availability of accurate noninvasive diagnostic tests may lead to treatment delays, also potentially contributing to poor outcomes.

Chest x-ray (CXR) offers a cost-effective [9], widely available, and noninvasive diagnostic tool for prompt PCP diagnosis in resource-limited health care settings. However, CXR features that are associated with laboratory-confirmed PCP and that can be used to discriminate PCP from other common respiratory infections in adults with HIV and to guide clinical decision-making in low-resource settings have only been explored in small observational studies, limiting clinical utility. A previous meta-analysis of CXR features associated with presumptive PCP and alternative diagnoses in adults with HIV in low- and middle-income countries was published in 2013 [6], highlighting the potential diagnostic value of CXR. However, in that review, clinical, not microbiological, definitions for PCP diagnosis were employed, and diagnostic performance of individual CXR features was not explored.

We conducted a systematic review and meta-analysis to characterize CXR changes in HIV-associated PCP. Our primary objectives were to describe the pattern and frequency of CXR changes in adults with HIV-associated laboratory-confirmed PCP and to compare these with CXR features from patients with non-PCP respiratory disease. As a secondary objective, we explored CXR features that correlate with clinical outcomes.

## METHODS

### Study Inclusion

Observational and interventional studies meeting eligibility criteria and published in peer-reviewed journals were included (Table 1). No language, clinical setting, or time restriction was applied. Studies enrolling mixed groups of people with or without HIV or employing clinical and/or laboratory-based definitions for PCP and non-PCP, without reporting disaggregated data in adults with HIV and laboratory-confirmed respiratory disease, were excluded. Studies where >20% of diagnoses in the group with non-PCP respiratory disease were not laboratory confirmed, without reporting disaggregated results within the laboratory-confirmed subgroup, were also excluded. Studies conducted before the availability of HIV serological testing but using the Centers for Disease Control and Prevention (CDC) definition of AIDS were included. In the absence of positive histology, visualization of typical lesions on bronchoscopy was regarded as confirmation of Kaposi's sarcoma.

**Table 1.** PICOT Criteria for Study Inclusion

P	Adults with HIV (≥15 years of age) undergoing investigation for any respiratory complaint
I	­Laboratory-confirmed PCP (*Pneumocystis jirovecii* detection using any microscopy or PCR laboratory method from any respiratory sample)
C	Non-PCP respiratory disease, as defined by (1) negative *Pneumocystis jirovecii* testing on any respiratory sample, and (2) alternative laboratory, histological, or bronchoscopy-confirmed (for at least 80% of enrolled non-PCP respiratory disease cohort, or if disaggregated results reported in laboratory-confirmed subgroup)
O	Pattern and frequency of CXR changes in adults with (1) PCP compared with (2) non-PCP respiratory disease; CXR features grouped into prespecified descriptive umbrella terms (Supplementary Table 1) [10–13]
T	No time restriction applied

Abbreviations: CXR, chest x-ray; PCP, *Pneumocystis* pneumonia; PCR, polymerase chain reaction.

### Literature Search Strategy

We searched Pubmed, Scopus, Web of Science, and EBSCO (including the Africa Wide and CINAHL databases) on November 12, 2022, with a repeat search on April 11, 2023. Our search strategy included 4 key components (*Pneumocystis* pneumonia, other respiratory infection[s], HIV, and chest radiography), without any language, time, clinical setting, or publication type limitations. Full search terms are included in Supplementary Data 1.

### Record Management and Data Collection

Records from the primary search were entered into COVIDence systematic review software [14], and duplicates were removed. Titles and abstracts were screened against the study eligibility criteria by N.W. and S.E., followed by review of full texts of potentially eligible studies for inclusion. Variables of interest were extracted to a Microsoft Excel spreadsheet by N.W. and verified by M.A. Study authors were contacted if data of interest were missing or unclear. Reference lists of included studies were searched to identify additional eligible studies. Included studies (all observational) were assessed using an adapted Newcastle-Ottawa scoring tool [15], with judgment of attrition and selection bias using the Cochrane Risk of Bias guidelines (Supplementary Data 2) [16]. S.W. was consulted for review of any discrepancies regarding study inclusion, data extraction, or study quality assessment.

### Data Analysis

Where heterogenous descriptive terminology was used across studies, CXR features were grouped using consensus umbrella terms (Supplementary Table 1) [10–13]. Data were pooled using a random-effects meta-analysis model with restricted maximum likelihood estimation. We computed proportions in the single-group meta-analysis (using metaprop [17] and the Freeman-Tukey arcsine transformation) and odds ratios (ORs) for the 2-group comparison (PCP vs non-PCP) with 95% CIs as measures of effect. Between-study heterogeneity was quantified using the *I*^2^ statistic. Where data from 2 or more studies were available, we performed prespecified subgroup analyses of studies reporting CXR features in adults with exclusive PCP (studies where codiagnoses were excluded or disaggregated results in adults with exclusive PCP were reported) as well as by study median CD4 count (<100 cells/mm^3^ or ≥100 cells/mm^3^) and time period of investigation (enrollment before or after 2005, reflecting the periods before and after wider global availability of potent combination ART and after transition from pentamidine to cotrimoxazole as PCP prophylaxis). Subanalysis by studies employing a systematic method for CXR review (radiologist involvement or predefined CXR review method if nonradiologists performing interpretation, with or without blinding to case clinical and microbiological information) vs an unspecified method for CXR interpretation was also conducted. Post hoc subgroup analysis on reviewer request was conducted by studies conducted in African vs non-African settings to explore possible interaction of higher background pulmonary tuberculosis (PTB) prevalence in Africa with radiological features. All meta-analyses were performed using Stata 17. The study protocol is registered on PROSPERO (ID CRD42023429073).

### Patient Consent

There were no factors necessitating patient consent for this review.

## RESULTS

### Characteristics of Included Studies

**Figure 1.**
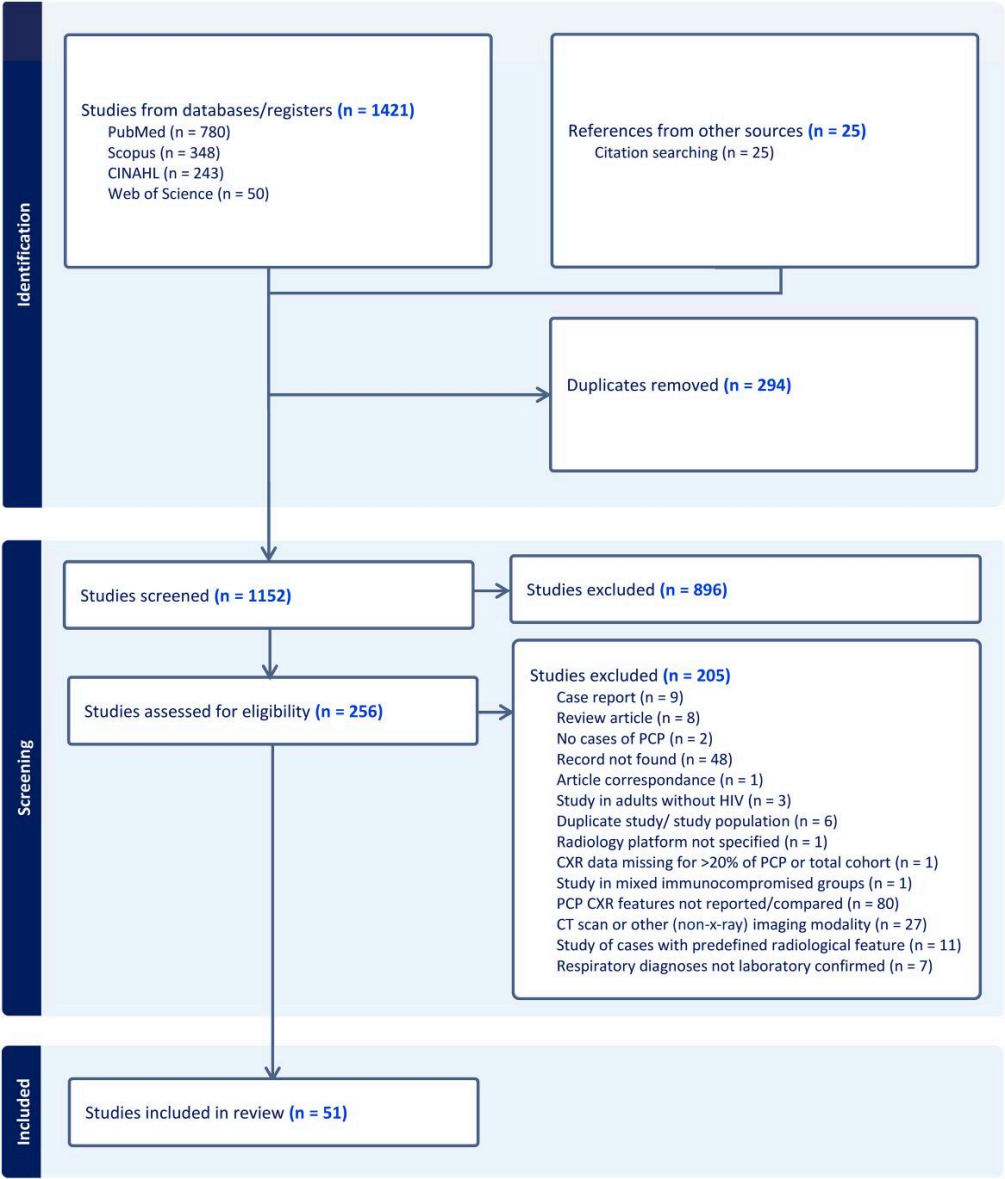
PRISMA diagram—flow of records from screening to final study inclusion. Abbreviations: CT, computed tomography; CXR, chest x-ray; PCP, *Pneumocystis* pneumonia; PRISMA, preferred reporting items for systematic reviews and meta-analyses.

A total of 1152 records were screened, with final inclusion of 51 studies, reporting CXR features from 2873 adults with HIV, including 1821 cases of PCP and 1052 cases of non-PCP respiratory disease (Figure 1). All studies were observational in design, with enrollment between 1981 and 2019 (Supplementary Table 2).

Twenty studies (1714 participants) provided data on CXR features among a mixed cohort with either laboratory-proven PCP (662 participants) or non-PCP respiratory disease (1052 participants), and 31 studies (1159 participants) reported CXR data from patients with PCP. Of this latter group, 6 studies enrolled cases of both PCP and non-PCP respiratory disease; in 4 studies, there was inadequate CXR information available to include data on non-PCP cases in the comparative 2-group meta-analysis [18–21], and in 2 studies the comparative arm was excluded because >20% of the non-PCP cases did not have a laboratory-confirmed diagnosis [22, 23]. One study reported radiological features from 38 patients with dual PCP and PTB infection [21].

Studies were conducted in Africa (10 studies, n = 636) [18, 24–32], North America (18 studies, n = 1301) [19–23, 33–45], South America (2 studies, n = 69) [46, 47], Central America (2 studies, n = 163) [48, 49], Europe (13 studies, n = 446) [13, 50–61], and Asia (6 studies, n = 258) [62–67]. Study settings included exclusive inpatients (29 studies, n = 1560; including 1 ICU study, n = 27) [36], mixed in- and outpatients (5 studies, n = 434), and postmortem (1 study, n = 69) [48]. The median or mean CD4 count was <100 cells/mm^3^ in 13 studies (n = 872) among the enrolled PCP or total cohort. Forty-one studies (2255 participants) conducted enrollment before 2005, and 10 studies (618 participants) conducted enrollment after 2005.

### CXR Features in Patients With PCP (Prevalence)

**Figure 2.**
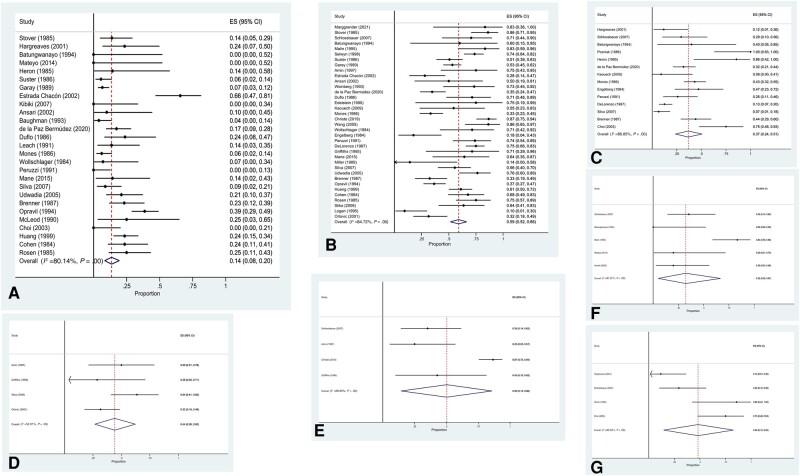
Radiological changes associated with PCP. Prevalence of normal CXR (A), interstitial (B), interstitial–alveolar (C), reticulonodular (D), reticular (E), and miliary. Abbreviations: CXR, chest x-ray; PCP, *Pneumocystis* pneumonia.

CXR was reported as normal in 14% (95% CI, 8%–20%) of PCP cases (27 studies, n = 1034; *I*^2^ = 80%) (Figure 2A). The most frequently reported CXR abnormalities included interstitial infiltrate in 59% (95% CI, 52%–66%; 36 studies, n = 1380; *I*^2^ = 85%), reticular infiltrate in 50% (95% CI, 14%–86%; 4 studies, n = 96; *I*^2^ = 90%), reticulonodular infiltrate in 44% (95% CI, 26%–62%; 4 studies, n = 79; *I*^2^ = 53%), interstitial–alveolar infiltrate in 37% (95% CI, 24%–51%; 14 studies, n = 467; *I*^2^ = 87%), ground-glass opacification in 48% (95% CI, 15%–83%; 4 studies, n = 57; *I*^2^ = 86%), and miliary changes in 32% (95% CI, 5%–67%; 5 studies, n = 55; *I*^2^ = 81%) (Figure 2B–G). Less frequent parenchymal changes included nodular infiltrate in 16% (95% CI, 5%–31%; 11 studies, n = 402; *I*^2^ = 89%), alveolar infiltrate in 15% (95% CI, 9%–21%; 30 studies, n = 1060; *I*^2^ = 82%), and consolidation in 10% (95% CI, 4%–18%; 19 studies, n = 423; *I*^2^ = 74%) (Supplementary Figures 1A–C).

Uncommon additional findings included cystic lesions in 8% (95% CI, 4%–13%; 3 studies, n = 180; *I*^2^ = 0%), pleural effusion or central lymphadenopathy in 4% (95% CI, 1%–7%; 20 studies, n = 649; *I*^2^ = 46%; and 18 studies, n = 608; *I*^2^ = 54%, respectively), cavitation in 3% (95% CI, 1%–6%; 14 studies, n = 443; *I*^2^ = 31%), and pneumothorax in 3% (95% CI, 0%–6%; 6 studies, n = 229; *I*^2^ = 0%) (Supplementary Figures 2A–E).

**Figure 3.**
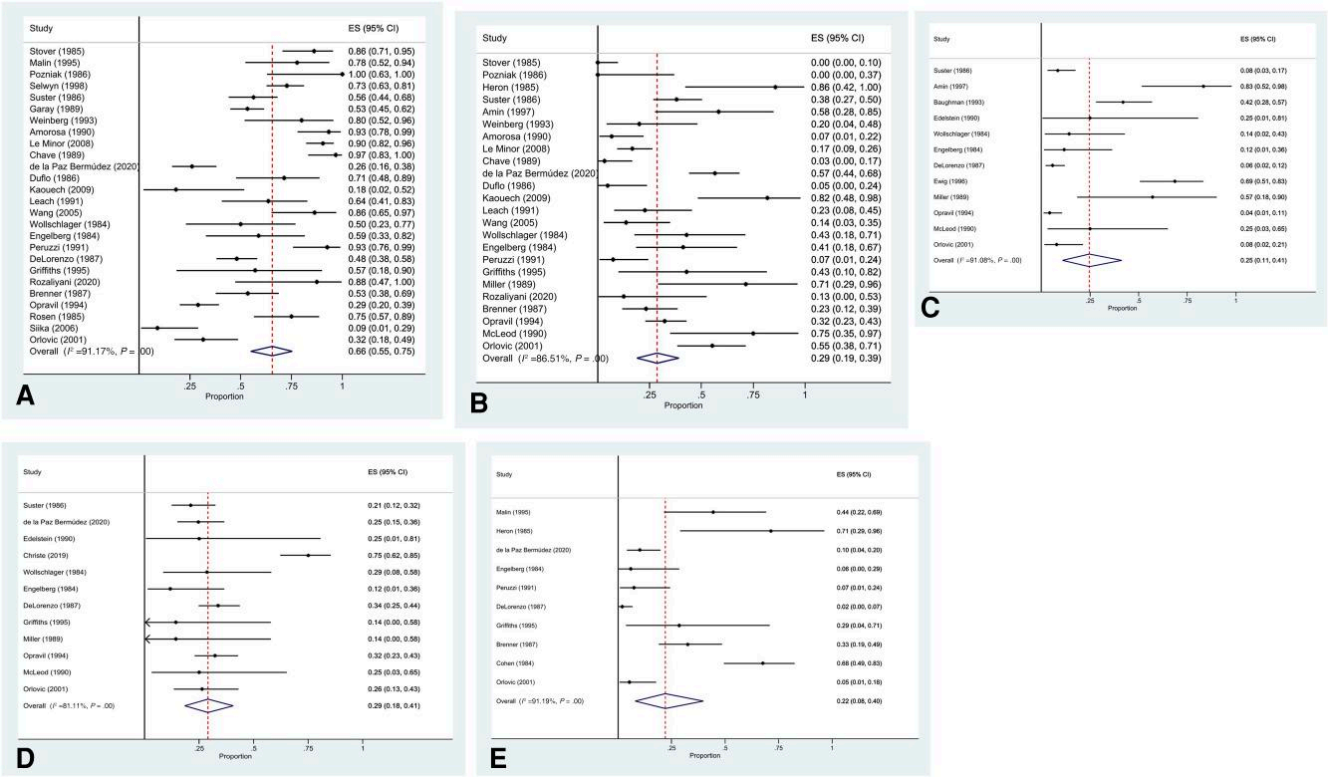
Distribution of CXR changes. Prevalence of diffuse CXR changes (A), focal changes (B), and upper zone (C), lower zone (D), and perihilar involvement (E). Abbreviation: CXR, chest x-ray.

Distribution of CXR changes was diffuse in 66% (95% CI, 55%–75%; 27 studies, n = 1152; *I*^2^ = 91%) and focal in 29% (95% CI, 19%–39%; 24 studies, n = 701; *I*^2^ = 87%). Frequency of lung zone involvement was similar, involving the upper zones in 25% (95% CI, 11%–41%; 12 studies, n = 453; *I*^2^ = 91%), lower zones in 29% (95% CI, 18%–41%; 12 studies, n = 453; *I*^2^ = 81%), and perihilar region in 22% (95% CI, 8%–40%; 10 studies, n = 364; *I*^2^ = 91%) (Figure 3A–E).

### Subgroup Analyses

Studies conducting a systematic radiology review, compared with studies where method of radiology review was not specified, reported lower rates of interstitial–alveolar disease (27%; 95% CI, 16%–39%; 10 studies, n = 421; vs 70%; 95% CI, 39%–95%; 4 studies, n = 46; group difference *P* = .008) and more upper zone involvement (38%; 95% CI, 12%–68%; 6 studies, n = 301; vs 8%; 95% CI, 4%–14%; 6 studies, n = 152; group difference *P* = .04) (Supplementary Figure 3A–B).

Compared with studies in which concomitant respiratory disease was not clearly excluded or results in PCP cases were not disaggregated, studies involving exclusive PCP cases reported a lower frequency of miliary infiltrates (19%; 95% CI, 1%–46%; 3 studies, n = 27; vs 62%; 95% CI, 43%–80%; 2 studies, n = 28; group difference *P* = .01), alveolar infiltrates (7%; 95% CI, 3%–13%; 16 studies, n = 564; vs 20%; 95% CI, 11%–32%; 14 studies, n = 496; group difference *P* = .02), and consolidation (4%; 95% CI, 1%–9%; 9 studies, n = 234; vs 16%; 95% CI, 4%–32%; 10 studies, n = 189; group difference *P* = .06). Pleural effusion was rare in cases with exclusive PCP (0%; 95% CI, 0%–2%; 10 studies, n = 298; vs 7% in adults with concomitant disease; 95% CI, 3%–12%; 10 studies, n = 351; group difference *P* = .00) (Supplementary Figures 4A–D).

Diffuse CXR changes were reported with higher frequency in studies with a median CD4 count <100 cells/mm^3^ (66%; 95% CI, 41%–87%; 8 studies, n = 411), compared with 36% (95% CI, 26%–47%; 2 studies, n = 87) in studies with a median CD4 count ≥100 cells/mm^3^ (group difference *P* = .02) (Supplementary Figure 5). There were no other significant interactions between median study CD4 count or time period of enrollment and reported CXR features. Studies from African settings reported higher prevalence of interstitial–alveolar disease, consolidation, pleural effusion, and diffuse CXR changes in PCP cases (Supplementary Figure 6A–E); these differences lost significance when restricting the analysis to studies enrolling exclusive PCP cases.

### CXR Features Associated With PCP Compared With Non-PCP Cases

Twenty studies providing CXR information from 1714 participants, including 662 cases with PCP and 1052 cases with non-PCP respiratory disease, were analyzed. Three CXR features were associated with PCP (Supplementary Figure 7A–C): interstitial infiltrate (OR, 2.3; 95% CI, 1.4–3.9; 12 studies, n = 1040; *I*^2^ = 60%), interstitial–alveolar infiltrate (OR, 10.2; 95% CI, 3.2–32.4; 4 studies, n = 138; *I*^2^ = 0%), and diffuse CXR changes (OR, 7.3; 95% CI, 2.7–20.2; 10 studies, n = 979; *I*^2^ = 87%). Presence of any infiltrate was also associated with an increased odds of PCP but with poor precision (OR, 11.5; 95% CI, 1.4–95.5; 3 studies, n = 313; *I*^2^ = 88%) (Supplementary Figure 7D).

The following CXR features occurred less frequently in PCP cases (Supplementary Figure 8A–E): alveolar infiltrate (OR, 0.1; 95% CI, 0.1–0.3; 12 studies, n = 844; *I*^2^ = 55%), consolidation (OR, 0.1; 95% CI, 0.04–0.3; 10 studies, n = 695; *I*^2^ = 59%), pleural effusion (OR, 0.5; 95% CI, 0.3–0.8; 12 studies, n = 904; *I*^2^ = 0%), central lymphadenopathy (OR, 0.3; 95% CI, 0.1–0.8; 10 studies, n = 913; *I*^2^ = 57%), and focal CXR changes (OR, 0.11; 95% CI, 0.03–0.5; 9 studies, n = 549; *I*^2^ = 83%). There were also lower odds of cavitation in PCP cases, although this did not reach statistical significance (OR, 0.5; 95% CI, 0.2–1.1; 8 studies, n = 579; *I*^2^ = 0%) (Supplementary Figure 9). A normal CXR, nodular infiltrate, or miliary infiltrate was not predictive of either PCP or non-PCP respiratory disease (Table 2; Supplementary Figure 10A–C).

**Table 2.** CXR Changes in PCP vs Non-PCP Respiratory Disease

CXR Changes^a^	No. of Studies (No. of Cases)	OR	95% CI	*I* ^2^ (%)
Associated with PCP				
Any infiltrate	3 (313)	11.5	1.4–95.5	88
Interstitial infiltrate	12 (1040)	2.3	1.4–3.9	60^b^
Interstitial–alveolar infiltrate	4 (138)	10.2	3.2–32.4	0^b^
Diffuse CXR changes	10 (979)	7.3	2.7–20.2	71
Associated with non-PCP respiratory disease				
Alveolar	12 (844)	0.1	0.1–0.3	55
Alveolar: consolidation	10 (695)	0.1	0.0–0.3	59
Pleural effusion	12 (904)	0.5	0.3–0.8	0^b^
Central lymphadenopathy	10 (913)	0.3	0.1–0.8	57^b^
Focal CXR changes	9 (549)	0.1	0.0–0.5	83^b^
No association				
Normal CXR	9 (800)	1.7	0.5–5.4	70
Interstitial–nodular infiltrate	7 (679)	0.7	0.2–2.2	69
Interstitial–nodular, miliary infiltrate	5 (354)	1.4	0.5–3.9	47
Cavitation	7 (419)	0.64	0.3–1.7	0

Abbreviations: CXR, chest x-ray; OR, odds ratio; PCP, *Pneumocystis* pneumonia.

^a^Insufficient data to conduct a 2-group meta-analysis on the following features: reticular infiltrate (2 studies, n = 97) [50, 53], reticulonodular infiltrate (1 study, n = 35) [50], ground-glass opacification (2 studies, n = 74) [53, 58], multilobar (1 study, n = 35) [50], bilateral (2 studies, n = 81) [24, 58], upper zone (2 studies, n = 131) [39, 50], lower zone (1 study, n = 96) [39], perihilar (2 studies, n = 69) [29, 58], involvement on CXR, pneumothorax, cysts or bullae (no studies), solitary nodules (1 study, n = 64) [41], bronchiectasis, collapse, bronchial thickening (all 1 study, n = 35) [50].

^b^Interactions on subgroup analyses outlined in Supplementary Figure 9.

There were no significant interactions observed on subgroup analysis, except for alveolar infiltrate, which did not distinguish PCP from non-PCP respiratory disease in studies among African populations (Supplementary Figures 11 and 12).

### Prognostic CXR Indicators in PCP Cases

Four small studies documenting radiographic severity, defined as new interstitial and alveolar infiltrates with or without increasing zone involvement, found significant associations between CXR progression and a higher fungal burden on microscopy (n = 81) [35], elevated lactate dehydrogenase (a marker of lung injury, n = 93) [51], and hypoxia (n = 136) [43, 51]. One study (n = 93) showed a correlation between PCP mortality at 3 weeks and severity of CXR infiltrates [51]. Another study (n = 43) reported longer median survival in those with normal to mild (8 months) vs more severe radiographic abnormalities (2 months), but this association was not statistically significant after adjustment for hypoxia [43]. An ICU-based study (n = 27) showed progression of infiltrates on serial CXRs over time, rather than baseline radiographic severity, to predict survival vs nonsurvival among critically ill patients with HIV-associated PCP [36].

### Study Quality

**Figure 4.**
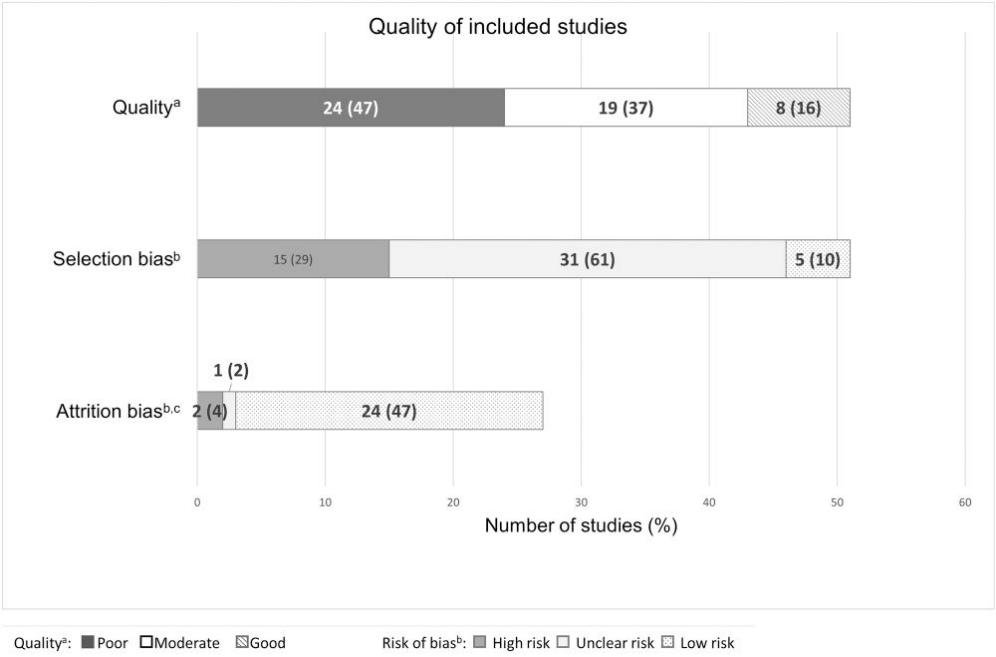
Quality of, and risk of bias in, 51 included studies. ^a^Assessed using an adapted Newcastle-Ottawa score. ^b^Assessed according to Cochrane Risk of Bias tool. ^c^Attrition bias not applicable in 24 noncohort studies.

All included studies were observational, with a systematic method applied to CXR review in 27 studies (n = 1610), radiologist interpretation of CXRs in 24 of these studies, and blinding of radiologists to clinical and microbiological data in 12 studies. Using the adapted Newcastle-Ottawa score, 24 studies (47%) were assessed to be poor quality (Figure 4;Supplementary Table 3). Fifteen studies (29%) were at high risk, and 31 studies (61%) were at unclear risk for selection bias, reporting CXR features in highly selected cohorts undergoing investigation for PCP, often after exclusion of smear-positive pulmonary tuberculosis (5 studies) [18, 25, 26, 29, 62], nonresponse to initial antibiotic therapy (5 studies) [24, 25, 28, 47, 52], or utilizing bronchoscopy for respiratory specimen sampling (23 studies), thereby excluding severely ill or hypoxic patients.

## DISCUSSION

This systematic review and meta-analysis provides insights into the patterns and distribution of CXR abnormalities observed among adults with HIV-associated PCP and illustrates specific CXR features that may aid in the differentiation of PCP from non-PCP respiratory disease. Interstitial infiltrate was the most frequent pattern reported among patients with confirmed PCP (59% of cases), most commonly in a reticular, reticulonodular, or interstitial–alveolar (including ground-glass) pattern. This observation, together with the high frequency of diffuse CXR involvement (reported in two-thirds of cases), reflects the pathophysiology of PCP in advanced HIV whereby *Pneumocystis jirovecii* induces widespread inflammation at the interstitial and alveolar–epithelial junction [68]. Alveolar infiltrates and consolidation were less frequently observed (15% and 10%, respectively), with cystic lesions, pleural effusions, central lymphadenopathy, cavitation, and pneumothorax reported infrequently.

Rather than inferring a pathognomonic cystic macroscopic lung injury, the term *Pneumocystis* evolved from early microbiological observations of fungal trophism for and adherence to alveolar epithelial cells (pneumocytes) during infection, where it undergoes transition from the trophic to the cystic form, followed by propagation and inciting the host inflammatory response [68–70]. Alveolar pneumocyte tropism may explain why pleural involvement in PCP is unusual and, if seen, is typically in the context of advanced immunosuppression with extensive parenchymal or extrapulmonary disease [71–73]. Limited ability of *Pneumocystis jirovecii* to bind to visceral pleura mesothelial cells has also been suggested as an additional explanation for the low frequency of pleural effusions in PCP [72]. Central thoracic lymphadenopathy in PCP tends to be less marked compared with that seen with other HIV-associated pathologies (particularly tuberculosis, fungal infections, and malignancy) [74, 75], but the low prevalence in our review may also reflect the lower sensitivity of CXR for detecting mild central lymphadenopathy compared with computed tomography (CT) scan [76, 77].

Historically, pneumothorax in patients with PCP had been linked to progressive upper zone fibrocystic disease in patients with pentamidine prophylaxis failure [78], but is also postulated to be a consequence of the exuberant host inflammatory response to *Pneumocystis jirovecii* reducing alveolar surfactant, rendering the lungs stiff and noncompliant and at risk of alveoli rupture spontaneously or with mechanical ventilation [79]. Previous commentaries that reported pneumothorax as a frequent complication of PCP may reflect a bias of retrospective reviews of cases of HIV-associated pneumothorax [80, 81] that may over-represent true overall prevalence among all cases of PCP. However, the low frequency of pneumothorax and cystic changes in our analysis may also reflect bias in inclusion of studies chiefly reporting CXR features on admission; only 2 included studies [44, 45] provided data on serial radiological reviews and reported development of new cystic lesions (6%), spontaneous pneumothorax (6%), procedure- or ventilation-related pneumothorax (14%), or recurrent pneumothorax after 1–5 months of follow-up.

On comparative analysis, the presence of interstitial infiltrate, interstitial–alveolar infiltrate, and diffuse CXR changes indicated a higher likelihood of PCP, while alveolar infiltrate, consolidation, pleural effusion, central lymphadenopathy, and focal changes were more indicative of non-PCP respiratory disease. These latter abnormalities are typical radiological features of bacterial pneumonia and pulmonary tuberculosis, common conditions in people with advanced HIV [10, 82]. Isolated pleural effusion, thoracic lymphadenopathy, or focal alveolar infiltrates strongly suggest an alternative diagnosis and may be helpful diagnostic tools for excluding HIV-associated PCP, particularly in combination with other clinical information [83]. In subgroup analysis, the absence of negative correlation between alveolar changes and PCP in studies from African populations may reflect differences in PCP disease phenotype or coinfection, such as TB.

In contrast, viral pneumonia may cause diffuse interstitial involvement indistinguishable from PCP [84]. When evaluating patients for PCP, the context of the high numbers of viral pneumonia cases seen with the recent coronavirus disease 2019 (COVID-19) pandemic and seasonal influenza is therefore challenging. A systematic review highlighted the propensity for COVID-19 to cause a pattern of peripheral and lower zone ground-glass opacification on CT of the chest [85], but in the absence of more specific radiological differentiators, testing for HIV should remain a priority to identify patients at risk of PCP [86, 87]. Cytomegalovirus (CMV) is a frequently isolated co-pathogen in patients with HIV-associated PCP [1], although the clinical significance is unknown. Three small studies in this review included a subgroup of patients with CMV and PCP coinfection with indistinguishable CXR changes from those with PCP alone [20, 38, 39]. Two additional studies enrolling adults with PCP and concomitant viral pneumonia did not report subgroup radiological features to allow comparison [22, 60].

In our review, studies enrolling patients with a lower CD4 count (mean <100 cells/mm^3^) reported a higher frequency of diffuse disease (66%) compared with mean CD4 count ≥100 cells/mm^3^ (36%). The extent of immune suppression strongly influences the radiological manifestation of HIV-associated pneumonias, including tuberculosis [88–90]. There are well-described differences in the pathophysiology, clinical features, and radiologic manifestations between HIV-associated PCP and HIV-negative PCP, driven by a more intense inflammatory response in the latter group [91, 92]. In CT studies enrolling adults with HIV- and non-HIV-associated PCP, focal consolidation and alveolar infiltrates were more commonly seen in non-HIV- compared with HIV-associated PCP [13, 93].

Several small studies have investigated the potential prognostic role of CXR in PCP [35, 43, 51], offering limited evidence that radiographic severity is associated with higher fungal burden, higher lactate dehydrogenase, worsening hypoxia, and increased mortality. Clinical studies have consistently shown that degree of hypoxia and elevated markers of inflammation or tissue injury are associated with poor outcome in PCP [5, 7, 8, 94]. The relationship between CXR involvement and clinical outcomes in PCP is confounded by concomitant respiratory disease. For example, PCP and tuberculosis coinfection was shown in a recent study to correlate with increased risk of mortality [8]. A limitation of studies investigating the prognostic value of CXR in PCP is that the interaction of respiratory coinfections with CXR changes and PCP outcomes was not explored.

This review has several additional limitations. First, we used consensus umbrella terms [10–12] to allow for grouping of CXR changes where heterogenous terminology was used across different studies; although terms were descriptive and took into account evolving international radiological definitions, information bias may have been introduced. Second, included studies were small and frequently enrolled select cohorts of patients. In particular, 2 subgroups of patients were not well represented in our review: first, acutely unwell and hypoxic patients not able to tolerate bronchoscopic evaluation, and hence excluded from bronchoscopy-based studies (in 23 out of 51 studies), and second, patients with “probable” PCP, with a compatible clinical syndrome but negative laboratory studies, who likely represent a specific radiological and clinical phenotype. However, a strength of our analysis was also that inclusion of studies reporting cases with a laboratory-confirmed PCP diagnosis (using positive PCR or microscopy) improved the specificity of findings, although both methods have imperfect accuracy and performance characteristics have changed over time. In aiming to explore the clinical utility of CXR as a tool for prompt PCP recognition among heterogenous HIV-related respiratory presentations, we grouped non-PCP pathologies into a single comparator group, rather than conducting an analysis across each individual diagnosis, to strengthen the PCP vs non-PCP comparative analysis. Furthermore, many of the larger comparative studies [26, 30, 34, 62] did not disaggregate results by specific non-PCP diagnosis. Lastly, nearly half of the included studies were assessed, using the Newcastle-Ottawa score, to be of poor quality. Although only small differences were found on subanalysis by method employed for CXR interpretation, limited radiologist involvement (specified in 47% of studies, with an otherwise systematic method for CXR review specified in 53% and clearly reported radiological definitions in 63% of studies), with a large contribution from nonexpert readers, may have compromised the accuracy of reported findings [95].

In conclusion, this systematic review and meta-analysis illustrates specific radiologic features in HIV-associated PCP supporting a central role in diagnoses. Although the radiological manifestations are diverse, certain abnormalities including interstitial infiltrate, with or without alveolar and ground-glass involvement, are highly predictive of PCP. In contrast, consolidation, pleural effusion, and central lymphadenopathy are not associated with PCP and should prompt investigation for alternative, or coexistent, pathologies such as tuberculosis or bacterial pneumonia. Radiologic features with high discriminatory value can be leveraged for use in standardized and systematic radiological tools to enhance the clinical utility of CXR for diagnosis of PCP. Ultimately, this approach could be incorporated into low-cost and accessible clinical prediction tools, including evolving artificial intelligence–assisted CXR reading software [96, 97], which may offer particular value in resource-limited settings where radiologists are not available, to improve recognition of this common and serious respiratory infection.

## Supplementary Material

ofae146_Supplementary_Data

## Supplementary Data

Supplementary materials are available at *Open Forum Infectious Diseases* online. Consisting of data provided by the authors to benefit the reader, the posted materials are not copyedited and are the sole responsibility of the authors, so questions or comments should be addressed to the corresponding author.
