# Supplementary material for: Chest X-ray Features of HIV-Associated Pneumocystis Pneumonia (PCP) in Adults: A Systematic Review and Meta-analysis
Source: Open Forum Infect Dis. 2024 Mar 18;11(4):ofae146. doi: 10.1093/ofid/ofae146 (PMC11020241; doi:10.1093/ofid/ofae146)
Supplement: ofae146_Supplementary_Data [file ofae146_supplementary_data.docx]

**Supplementary material**

**Tables**

Table S1. Terminology used for grouping of CXR features reported across studies, using consensus umbrella terms.

Table S2A. Summary of included studies.

Table S2B. Study radiology definitions and approach to chest X-ray review.

Table S3. Quality of, and risk of bias in, 51 included studies.

**Figures**

Figure S1. Prevalence of nodular (A) and alveolar infiltrates (B) and consolidation (C) in adults with HIV-associated PCP.

Figure S2. Prevalence of cystic changes (A), pleural effusion (B), central lymphadenopathy (C), cavitation (D) and pneumothorax (E) in in adults with HIV-associated PCP.

Figure S3. Prevalence of interstitial-alveolar disease (A) and upper zone disease (B) in adults with HIV-associated PCP, stratified by studies conducting a systematic versus unspecified radiology review.

Figure S4. Prevalence of miliary infiltrate (A), alveolar infiltrate (B), consolidation (C) and pleural effusion (D) in in adults with HIV-associated PCP, stratified by studies reporting radiological features in exclusive PCP cases.

Figure S5. Prevalence of diffuse CXR changes in adults with PCP, stratified by median study CD4 count.

Figure S6. Prevalence of interstitial-alveolar infiltrate (A), consolidation (B), pleural effusion (C), diffuse CXR changes (D) and focal CXR changes (E) in adults with PCP, stratified by African versus non-African study setting.

Figure S7. CXR features predictive of PCP versus non-PCP respiratory disease in adults with HIV: interstitial infiltrate (A), interstitial-alveolar infiltrate (B), diffuse CXR changes (C), and any infiltrate (D)

Figure S8. CXR features that correlate with non-PCP respiratory disease in adults with HIV: alveolar infiltrate (A), consolidation (B), pleural effusion (C), central lymphadenopathy (D), focal CXR changes (E).

Figure S9. Odds of PCP versus non-PCP respiratory disease in adults with HIV with cavitation on CXR

Figure S10. Odds of PCP versus non-PCP respiratory disease in adults with HIV with a normal CXR (A), interstitial-nodular infiltrate (B) or miliary infiltrate (C).

Figure S11. Odds of PCP versus non-PCP respiratory disease in adults with HIV with (A) interstitial infiltrate, stratified by median study CD4 count (B) interstitial-alveolar infiltrate, stratified by systematic versus unspecified CXR review, (C) effusion, stratified by systematic versus unspecified CXR review, (D) central lymphadenopathy, stratified by time-period of enrolment, and (E) focal CXR changes, stratified by systematic versus unspecified CXR review.

**Appendices**

Appendix 1. Search terms used on Pubmed and adapted for use on other databases

Appendix 2. Study quality and risk of bias assessment tool

**Table S1. Terminology used for grouping of CXR features reported across studies, using consensus umbrella terms**^[1–4]^

| **Parenchymal** | | | | |
| --- | --- | --- | --- | --- |
| Review terms | | | | Definition/inclusion terms |
| Normal |  |  |  | Normal CXR |
| Parenchymal subcategories | Infiltrate not specified |  |  | Includes NS terms such as “parenchymal opacification”; and only if not included in other parenchymal patterns |
|  | Interstitial | Interstitial (total) |  | Total of all records noted to have interstitial, reticular, reticular-nodular, or nodular, and/or nodular-miliary features.  Also included in this category: any connective tissue involvement (axial, acinar or subpleural). |
|  |  | Reticular |  | Reticular: innumerable fine linear opacities, that by summation give net-like appearance |
|  |  | Reticulo-nodular |  |  |
|  |  | Nodular | Nodular (total) | Total reported with nodular and/or miliary changes |
|  |  |  | Nodular-miliary | Included in miliary category if reported as nodular with multiple diffuse uniform nodules ≤ 3mm |
|  | Interstitial-alveolar | Interstitial-alveolar (total) |  | Total of all records noted to have interstitial-alveolar and/or ground glass features |
|  |  | Ground glass |  | Where reported as ground-glass opacification. |
|  | Alveolar | Alveolar (total) |  | Total of all records noted to have consolidation and/or any other noted alveolar involvement, including acinar and/or non-specific airspace involvement |
|  |  | Consolidation |  | Consolidation and lobar shadowing included in this category |
| **Distribution of parenchymal changes** | | | | |
| Review terms | | | | Definition/inclusion terms |
| Localisation of parenchymal changes | Diffuse OR |  |  | As labelled by researchers, or if noted to involve all lobes of both lungs; or bilateral AND multilobar |
|  | Focal/patchy/ unilobar | Bilateral, if not included in diffuse |  |  |
|  |  | Unilateral |  |  |
|  | Upper zone involvement OR |  |  | Upper lobe, apices, upper lung |
|  | Mid-zone involvement OR |  |  | Middle lobe, lingula, mid-zone |
|  | Lower-zone involvement |  |  | Basal, lower lobe, lower zone. Some studies eg Andreas at al – basal included middle ® and lingula lobe |
|  | Perihilar OR |  |  |  |
|  | Peripheral OR |  |  |  |
|  | Perihilar and peripheral |  |  |  |
|  | Subpleural sparing |  |  |  |
| **Additional features**: | | | | |
| Captured as reported in studies (cavitation, pleural effusion, pneumothorax, central adenopathy (including hilar or mediastinal lymphadenopathy, solitary nodule(s), bronchiectasis, honey combing collapse/atelectasis, isolated cystic lesions (not associated with honeycombing, bronchiectasis or other structural disease), bullae (airspace > 1cm, thin walled (≤ 1mm) | | | | |
| **Other** | | | | |
| Note if score given for overall extent of disease, and how score calculated.  Note any prognostic associations with CXR or clinical features. | | | | |

**Table S2A. Summary of included studies.**

| Author (year)  Study period | Study question | Study design | Inclusion criteria | Exclusion criteria | Country | Setting (Inpatient/outpatient) *level of care only indicated for ICU study | PCP laboratory diagnosis | Total sample; ADULTS with HIV and laboratory-confirmed diagnosis and CXR features reported | Median/mean CD4 (IQR, or ±SD or range) | Number on CTX/other prophylaxis | Mean/median age (IQR, or ±SD or range) | Mean/median Pa02 (mmHg or kPa, IQR, or ±SD or range) or mean/median oxygen saturation (pulse oximetry, %) | Sample size: PCP and non-PCP respiratory disease | Sample size: PCP | Sample size: non-PCP respiratory disease |
| --- | --- | --- | --- | --- | --- | --- | --- | --- | --- | --- | --- | --- | --- | --- | --- |
| COMPARATIVE STUDIES | | | | | | | | | | | | | | | |
| Chave (1989) ^[5]^  Apr 1985 - Mar 1988 | Review of 54 episodes of pneumonia in patients with HV | Retrospective record review | Pneumonia noted on medical record review (new chest infiltrate associated with fever and respiratory symptoms), HIV positive | NS | Switzerland (Lausanne) | NS | Cysts in BAL fluid. Stain NS | 54 episodes; laboratory confirmed diagnoses with CXR data reported: n = 49 | NS | NS | NS | NS | 49 | 31 | 18 |
| Garay (1989) ^[6]^  NS | Prognostic indicators in the initial presentation of PCP | Prospective cohort | HIV positive, presenting with PCP as initial presentation of AIDS or newly dx HIV and pulmonary process other than PCP. | (1) PCP with co-infections (25 patients), (2) Liver function abnormalities | USA (New York) | Inpatient | BAL/ transbronchial biopsy: Giemsa/methanamine silver | 217; CXR data reported in: n = 191 | NS | NS | PCP: 33 ± 5. Non-PCP: 35 ± 3 | Mean Pa02: PCP: 73 ± 7, non-PCP: 78 ± 5 mmHg | 191 | 150 | 41 |
| Marggrander (2021) ^[7]^  Sep 2017 - Apr 2019 | Lung US vs CXR and CT for diagnosis of HIV-associated lung disease | Prospective | Hospitalised in department of infectious disease at study site, adults with HIV, with definite diagnosis of pulmonary disease | HIV neg or not consenting, lack or refusal of diagnostics needed for confirmed diagnosis | Germany (Frankfurt) | Inpatient | PCR + microscopy | 80 total; confirmed diagnosis with CXR n= 32 | 137.5 (IQR 37.7 - 330.7). PCP (n = 21) : 36 (18 - 47) | | 47 (IQ 40 - 52) | NS | 32 | 6 | 26 |
| Stover (1985) ^[8]^  NS, 4 yrs | Spectrum of pulmonary diseases associated with AIDS | Prospective | Diagnosis of AIDS (CDC criteria), with pulmonary disease on admission or developed during the study | NS | USA (New York) | Inpatient | Cysts in bronchial fluid or lung tissue; microscopy - silver methenamine | 130; n = 87 with confirmed diagnosis and CXR reported | NS | NS | 37 (range 24 - 70) | NS | 98 | 36 | 62 |
| Batungwanayo (1994)^[9]^  Jan 1990 - Nov 1990 | Pulmonary disease associated with HIV in Kigali, Rwanda | Cross sectional | Patients referred to pulmonary division for bronchoscopy for pulmonary disease of undetermined aetiology: (1) any respiratory symptom, (2) any type of infiltrate on CXR or normal CXR with persistent unexplained respiratory signs/symptoms, (3) no response to empiric antibiotics (4) three sputum smear negative for MTB | NS | Rwanda (Kigali) | NS | Microscopy: methenamine silver | 111 investigated; CXR and confirmed respiratory diagnosis: n = 53 | NS | NS | NS | NS | 53 | 5 | 48 |
| Hargreaves (2001)^[10]^  Nov 1997 - Jun 1998 | PCP in patients registered with smear-negative PTB in Malawi | Prospective cohort | Patients registered for smear negative PTB (3 sputum samples negative, with non-response to antibiotics and with subsequent suspicious CXR). | Patients < 15 years | Malawi (Lilongwe) | Inpatient and outpatient (Inpatients - PCP 1/17 and non-PCP 17/169. Remainder outpatients) | Indirect IFA and PCR | 186 (352 registered for smear-neg PTB; investigation for PCP n = 186) | NS | NS | PCP: 35 (IQR: 33 - 39), non-PCP: 33 (28 - 40) | Sats: Resting - PCP: 95 (93 - 96), non-PCP: 96 (94 - 97) mmHg | 176 | 17 | 159 |
| Schlossbauer (2007)^[11]^  NS | CXR and CT features in patients with HIV-associated lung disease | Cross-sectional | Adults with HIV with pulmonary symptoms, referred to study site radiology department for imaging (CXR and CT) | NS | Germany (Munich) | Inpatient and outpatient | Stain not specified | 130; CXR reported in n = 57 | 225 (44% with CD4 < 200 cells/mms) | NS | 43.8 (range 22 - 77 yrs) | NS | 62 | 17 | 45 |
| Heron (1985)^[12]^  Aug 1983 - Oct 1984 | Radiographic features in patients with pulmonary manifestations of AIDS | Retrospective review | Retrospective review of 14 episodes of pulmonary pathology in 13 patients with AIDS; enrolment criteria not further reported | NS | United Kingdom (London) | Inpatient | Silver methenamine | 14; CXR features reported: n = 12 | NS | NS | 35 (range 25 - 41yrs) | NS | 12 | 7 | 5 |
| Le Minor (2008) ^[13]^  Sep 2002 - Oct 2004 | Causes of AFB sputum smear negative pneumonia in Asian and African HIV-infected adults | Cross-sectional | Age ≥ 18 yrs, at least 1 clinical sign of lung infection, new radiological diffuse or localised lung opacities or mediastinal LAD on CXR, HIV-positive, AFB smear negative x 1 | Not able to perform bronchoscopy (non-consent or too ill). Others NS. | Cambodia, Vietnam | NS | 5 cysts of *P.jirovecii* on microscopy of BAL; IFA staining | 255; n = 160 (PCP and non-PCP group) with CXR reported | CD4 ≤ 50 in 72/84 PCP patients (88%) and 55/76 non-PCP patients (74%) | 3/84 PCP patients (4%), 30/76 non-PCP patients (39%) | PCP group: age > 34 yrs in 40/84 (48%), non-PCP group: age > 34 yrs in 36/76 (47%) | PCP: Sa02 < 80% in 24/84 (29%) and < 90% in 34/84 (40%), Non-PCP: Sats < 80% in 2/76 (3%) and < 90% in 11/76 (14%) | 160 | 84 | 76 |
| Malin (1995)^[14]^  May 1992 - May 1993 | Prevalence of PCP in Zimbabwe | Prospective | HIV-positive, 18 - 65 years, acute diffuse pneumonia, unresponsive to antibiotics, three sputum smear negative for MTB, referred for bronchoscopy | <3 zones involved in CXR, or cavitatory changes | Zimbabwe (Harare) | Inpatient | *P. jirovecii* cysts and/or trophozoites were identified with three stains in all cases (methenamine-silver, toluidine blue 0, and Diff-Quik). + PCR | 64; CXR data available: n = 57 | PCP: 134 (range 5 - 355) | NS | In 64: 35.5 (range 24 - 50) | PCP: Pa02 < 75mmHg 11/13, and Pa02 < 50mmHg 6/13. In non-PCP Pa02 < 75mmHg 25/37, and Pa02 < 50mmHg 4/37 | 57 | 18 | 39 |
| Mateyo (2014) ^[15]^  NS | Pulmonary disease in adults with HIV in Zambia | Cross-sectional | Every 2nd pre-ART adult with HIV presenting for admission to the study hospital with CD4 < 200 cells/cm3 and pulmonary symptoms | NS | Zambia (Lusaka) | Inpatient | If sputum AFB neg or unable to expectorate sputum: bronchoscopy + microscopy for PCP. Stain NS. | 113 | 55 (21 - 75) | 20/113 (PCP: 0/5) | 34.9 (± 8.9) | Median sats: 92 (87.5 - 96.0). PCP sats < 85% in 2/5 | 113 | 5 | 108 |
| Pozniak (1986) ^[16]^  NS | Clinical and bronchoscopic diagnosis of suspected HIV-associated pneumonia | Prospective | Adults with HIV, symptoms of LRTI, abnormal CXR and resting hypoxia in room air or alveolar-arterial oxygen gradient ≥ 20mmHg. Patients with suspected PCP but not improving on CTX then underwent BAL or transbronchial biopsy. | NS | United Kingdom (London) | Inpatient | NS - BAL/transbronchial biopsy but laboratory method not reported | 11 | NS | NS | PCP: 38 (25 - 43), Bacterial pneumonia 36 (33 - 38) | Mean Pa02: PCP: 67 (45 - 73) mmHg. Bacterial pneumonia: 69 (64 - 74) | 11 | 8 | 3 |
| Selwyn (1998)^[17]^  March 1986 - July 1994 | Clinical predictors of PCP, bacterial pneumonia and PTB in patients with HIV | Retrospective (chart review) | (1) > 18 yrs, (2) Hospitalised during study period at the study site, (3) primary discharge diagnosis of PCP, PTB or community-acquired pneumonia | Cases required laboratory confirmation | USA (New York, Connecticut and Washington) | Inpatient | Presence of *P. jirovecii* cysts in stained specimens from induced sputum, bronchial lavage, or transbronchial biopsy. Stain not specified | 229 | PCP: 20 (3 - 48), PTB 96 (47 - 165), BP 192 (19 - 370) *difference p < 0.001 | < 20% |  | Median P02: PCP: 67 (57 - 79), BP: 79 (68 - 86), TB: 82 (75 - 101) | 229 | 99 | 130 |
| Suster (1986)^[18]^  Feb 1982 - Nov 1984 | Pulmonary manifestations of AIDS: review of 106 episodes | Retrospective review | All patients admitted to study institution with pulmonary disease and later proved to have AIDS, with clinical and radiological records available, with laboratory/pathology/clinically confirmed pulmonary disorder | NS; 8 patients excluded due to interpretative difficulties with CXRs | USA (New York) | Inpatient | Stain NS | 95; 106 episodes (11 individuals admitted twice over study period) | NS | NS | Range 20 - 60 yrs (mean 32.5) | NS | 96 | 71 | 25 |
| Amin (1997)^[19]^  Mar 1987 - Feb 1995 | Correlation between CXR abnormalities and diagnosis in patients with HIV and with acute respiratory symptoms | Retrospective, cross-sectional (record review) | Acute (< 2 weeks), community acquired respiratory episode, admitted as inpatient, HIV positive | Non-infective aetiologies (eg KS) | United Kingdom (London) | Inpatient | Microscopy of sputum and/or bronchoscopy sample; staining method not specified | 53; CXR and micro confirmation n = 35 | 40 (range 10 - 780) | Inhaled pentaminide; of 10 patients with UL consolidation and PCP, 3 received prophylaxis | 37 yrs (range 23 - 624 yrs) | NS | 35 | 12 | 23 |
| Amorosa (1990)^[20]^  1984 - 1988 | Radiological distinction of pyogenic infection from PCP | Retrospective (1984 - 1986; chart review), prospective (1986 - 1988) | HIV-1 infection and pulmonary disease; 30 patients (34 episodes) with pyogenic infection matched with 30 patients with PCP (matched by age, sex, risk factor) | No pathological confirmation | USA | NS | Microscopy (stain NS); BAL or open lung biopsy | 60 (across 64 episodes) | NS | Nil | NS | NS | 64 | 30 | 34 |
| Ansari (2002)^[21]^  Jul 1997 - Jun 1998 | Pathology and causes of death in patients with HIV in Botswana | Autopsy study + retrospective radiography review | Death before diagnosis found, deterioration in hospital and in those with respiratory disease (14% of all inpatient deaths) | NS | Botswana (Gaborone) | Inpatient | Grocott | 128 total; with HIV n = 104 | NS | NS | In 128: 36 (range 14 - 87). In PCP: 32 (21 - 45), PTB: 36 (16 - 69), Pneumonia: 36 (18 - 68) | NS | 69 | 10 | 59 |
| Estrada Chacón (2002)^[22]^  Jan 2001 - Jun 2001 | Radiological changes in adults with HIV and with acute respiratory infection | Retrospective, cross-sectional (record review) | (1) adults with HIV (2) acute respiratory infection during study period | NS | Cuba (Havana) | Inpatient | BAL; microscopy methods unclear | 94 | 79 patients with CD4 < 200 | NS | NS | NS | 94 | 32 | 62 |
| Kibiki (2007)^[23]^  NS | Aetiology and outcome of pulmonary infections in Tanzanian people with HIV | Prospective; outcome at DC or for outpatients at 4/52 follow up | Recruitment at bronchoscopy centre: adults with HIV presenting with cough or any other respiratory complaint to study centre, referred from bronchoscopy by managing clinician (no aetiology found or non-response to empiric Rx) | Pregnant. Oxygen sats < 90% on NP02 | Tanzania (Moshi) | Inpatient and outpatient | BAL + PCP: giemsa, gomori methenamine silver (GMS), IF test, PCR. HHV8: PCR | 120; CXR data with micro-confirmed diagnosis in: n = 80 | 47 (IQR 14 - 91) in 71 patients with confirmed aetiology. In 9 PCP group: CD4 median 26 (IQR 7 - 91) | NS | 39 (SD ± 10) | Sats: median 92 ± 8 | 89 | 9 | 80 |
| Weinberg (1993)^[24]^  June 1998 - May 1989 | Respiratory complications in Brazilian patients infected with HIV | Prospective (15 patients), retrospective (20 patients) | Fever/dyspnoea and cough with abnormal CXR, undergoing pulmonary investigations (sputum induction, or if inconclusive/clinical worsening on treatment, sent for bronchoscopy) | NS | Brazil (Săo Paulo) | Inpatient | Identification of cysts on toluidine blue smear (sputum/BAL) or Grocott's tissue stain | 35 | NS | NS | 34.11 (22 - 52) | NS | 24 | 15 | 9 |
| DESCRIPTIVE STUDIES | | | | | | | | | | | | | | | |
| Baughman (1993)^[25]^  NS; 9 months | Lobar distribution of radiological disease and *P.jirovecii* cyst density (from BAL) in patients with PCP | Cross-sectional | Adults with HIV presenting with pulmonary symptoms | NS | USA (Cincinatti) | NS | Wright-Giemsa | 52 | NS | NS | NS | NS | 50 | 50 | NA |
| Christe (2019) ^[4]^  2005 - 2012 | Comparison of CXR and CT findings in HIV-asssociated versus renal-transplant associated PCP | Retrospective (cross-sectional registry analysis) | Databases screened from 2005 - 2012 for PCP cases meeting predetermined definition for confirmed PCP | NS | Switzerland | NS | Cytology and/or histology | 60 | 79 ( 3 - 436) | NS | 43 (25 - 76) | NS | 60 | 60 | NA |
| de la Paz Bermúdez (2020)^[26]^  Jan 1996 - Jan 2014 | Radiological findings of PCP in Cuban deceased HIV/AIDS patients | Autopsy study; retrospective radiography and clinical review | (1) Cuban, HIV positive, deceased with (2) positive *P. jirovecii* microscopy (positive mortem lung biopsy) (3) CXR available | NS | Cuba | Autopsy | Microscopy of lung biopsy | 69 | 149 (SD ± 198) | NS | 38 (SD ± 9.9) | NS | 69 | 69 | NA |
| Duflo (1986)^[27]^  June 1982 - Dec 1984 | Retrospective review of 21 cases of PCP | Retrospective cohort (folder review, in-hospital outcome reported) | All AIDS-associated PCP seen at the Tropical Medicine Hospital for the study period | AIDS without PCP | France (Paris) | Inpatient | Microscopy (silver stain and others) | 21 | NS | NS | NS | Mean Pa02: 61mmHg | 21 | 21 | NA |
| Edelstein (1990)^[28]^  June 1987 - Dec 1989 | Atypical presentations of PCP in patients receiving inhaled petamidine prophylaxis | Retrospective cohort (folder review) | Patients with recurrent episode of PCP on PCP prophylaxis | NS | USA (Martinez) | Inpatient | NS | 4 | NS | All on inhaled pentamidine, range 8 - 25 monhs | 4 cases: 29, 31, 34, 35 | NS | 4 | 4 | NA |
| Engelberg (1984) ^[29]^  Mar 1981 - Oct 1982 | Clinical features of PCP in AIDS | Retrospective | All cases of PCP seen at the study hospital during the study period retrospectively reviewed | NS | USA (New York) | Inpatient | Gomori's methenamine silver stain, hematoxylinphloxine- safran stain, and Kinyoun's acid-fast stain. Bronchoscopy with transbronchial biopsy or BAL | 17 | NS | NS | 41 yrs (range 28 - 56yrs) | Pa02 range 33 - 84 mmHg | 17 | 17 | NA |
| Kaouech (2009) ^[30]^  Apr 2005 - Dec 2007 | PCR vs microscopy + value of CXR for PCP diagnosis | Retrospective cohort; outcome data captured | Review of clinical data for patients with BAL/ TA/ sputum/pleural fluid available for testing. adults with HIV or immunocompromised (disaggregated data available) | NS | Tunisia (Tunis) | NS | Conventional PCR and staining techniques (Gomori-Grocott, May-Grünwald Giemsa). BAL/ TA/ sputum/pleural fluid available for testing | 54 | NS | NS | Range for 10 PCP cases: 35 - 57 years | NS | 11 | 11 | NA |
| Leach (1991)^[31]^  NS | Value of CXR and DTPA scan for PCP diagnosis and to guide selection of patients for bronchoscopy | Prospective | Adults with HIV, presenting with fever, cough and breathlessness | NS | United Kingdom (London) | Inpatient and outpatient | Induced sputum ± bronchoscopy if diagnosis not clear after DTPA scan + CXR + if failed response to empiric cotrimoxazole treatment | 72 | NS | NS | 72 pt: 35 (rage 19 - 61) | Mean Pa02 PCP: 8.3 ± 0.5kPa (range 4.7 - 14.7) | 22 | 22 | NA |
| Mones (1986)^[32]^  1981 - 1984 | CXR correlates in bronchoscopy positive cases of PCP | Cross-sectional | All patients with PCP diagnosed on bronchoscopy at study site between 1981 - 1984 | NS | USA (Miami) | NS | Diagnosis confirmed on bronchoscopy, (transbronchial biopsy or lavage/brushings); microscopy method - Grocott's modification of GMS | 95 | NS | NS | med 32 (range 21 - 60yrs) | NS | 81 | 81 | NA |
| Peruzzi (1991)^[33]^  Jan 1985 - Mar 1989 | ICU experience of PCP cases and predictors of outcome in PCP patients admitted to ICU | Retrospective; medical record review | All patients with HIV admitted with PCP to study site ICU from Jan 1985 - March 1989 | Absence of microscopy-identified *P.jirovecii* | USA (Chicago) | Inpatient; ICU | Sputum induction or BAL if not identified after induction x 2. GMS, toluidine blue O and giemsa staining. | 27 | NS | 27-Jan | Survivors: 32.9 (± 7.8), non-survivors: 36.6 ± 9.7) | Mean PF: Survivors mean PF 293 (± 122), non-survivors PF 249 (± 53); Mean Pa02: Survivors mean Pa02 62 kPa± 26, non-survivors Pa02 57 kPa ± 14 | 27 | 27 | NA |
| Wang (2005)^[34]^  Jan 1992 - Oct 2004 | Clinical characteristics and outcome of PCP in patients with AIDS | Retrospective cohort | 22 patients with microscopy-confirmed PCP treated at study hospital | NS | China (Beijing) | Inpatient | Toluidine blue, silver stain or giemsa staining for cysts/trophoziotes | 22 | Range 3 - 148 cells/mm3 (90% CD4 < 100 cells/mm3) | NS | 35.0 ± 9.4 | Pa02 < 60mmHg: 14/22 | 22 | 22 | NA |
| Wollschlager (1984)^[35]^  1981 - Jul 1983 | Pulmonary manifestations of AIDS | Retrospective (medical record and autopsy review) | Record review of all patients diagnosed with AIDS and seen at the study hospital during the study period; included 14 patients with PCP and 1 with necrotizing pneumonia | NS | USA (New York) | Inpatient | Isolation of cysts/trophozoites on sputum, open lung biopsy, transbronchial biopsy (n = 10) or autopsy tissue staining (n = 4) | 15 | NS | NS | 32 (23 - 43yrs) | Arterial-alveolar gradient range: 43 - 76 | 14 | 14 | NA |
| Brenner (1987)^[36]^  Jan 1983 - Apr 1986 | Prognostic factors in patients with AIDS-associated PCP | Prospective | All patients meeting AIDS criteria with pulmonary symptoms and/or abnormal CXR and referred for bronchoscopy within 48 hrs of antibiotic therapy | Excluded patients with incomplete data available (n = 12) | USA (Bethesda) | NS | Modified toluidine blue O stain: BAL, transbronchial biopsy, or open lung biopsy. | 55 | NS | NS | NS | NS | 43 | 43 | NA |
| Choi (2003)^[37]^  1996 - 2002 | Comparison of PCP between adults with and without HIV | Retrospective (folder review) | Admitted to study hospital, immunosuppressed (HIV or non-HIV; disaggregated data), and diagnosed with PCP between 1996 - 2002 | NS | Korea (Seoul) | Inpatient | Micrscopy, stain NS | 16 | NS | 0 | 41 ± 9.6 | 61 ± 17 | 16 | 16 | NA |
| DeLorenzo (1987)^[38]^  1981- 1985 | Roentgenographic patterns of PCP in patients with AIDS | Prospective cohort | Fulfilling CDC criteria for AIDS and referred to pulmonary department with pulmonary symptoms for investigation | Patients with other respiratory diagnoses in addition to PCP | USA (New York) | Inpatient | Bronschoscopy and lung biopsy ± BAL: giemsa + methenamine silver stain | 104 | NS | NS | NS | NS | 104 | 104 | NA |
| Ewig (1996)^[39]^  May 1989 - June 1995 | Effect of long-term primary aerosolized pentamidine prophylaxis on breakthrough PCP | Prospective cohort (1990 - 1995) and retrospective cohort (record review, 1989) | All patients with breakthrough PCP, derived from population receiving aerosolized pentamidine prophylaxis at study institution (indication: CD4 < 200 or AIDS-defining illness), and 30 cases of PCP in patients not on prophylaxis | NS | Germany (Bonn) | Inpatient | BAL/ transbronchial biopsy: Giemsa/grocott stain | 50; CXR results reported: n = 35 | 39 ± 38 | 20/50 on aerosolized pentamidine prophylaxis; 6/50 early failure (≤ 12 months of therapy) and 14/20 late failure (> 12 months). | No prophylaxis: 39 ± 12, failures: 36 ± 10 | Mean Pa02: No prophylaxis: 6.0 ± 2.3 kPa, Failure: 5.7 ± 2.0 kPa | 35 | 35 | NA |
| Griffiths (1995)^[40]^  NS | Causes of interstitial pneumonitis in people with HIV | Retrospective (record review, cross sectional) | Dyspnoea ± cough and CXR with diffuse infiltrates | NS | United Kingdom (London) | Inpatient | Induced sputum, transbronchial biopsy or lung biopsy | 7 | 62/42/17 | NS | 41 | Range Pa02: 5.9 - 13.1 kPa | 7 | 7 | NA |
| Mane (2015)^[41]^  NS | PCP and DHPS/DHFR mutations in people with HIV in India | Prospective cross sectional | Patients with HIV attending tertiary hospital with symptoms of LRTI > 7days | NS | India (Maharashtra) | NS | Grocott’s Gomori methenamine silver (GMS) stain + PCR | 111 | All data here for PCP cases (n = 14). CD4 123 (range 48 - 239) | 6/14 | 38 (Range 24 - 62) | NS | 14 | 14 | NA |
| SILVA (2007) ^[42]^  Jan 2001 - Dec 2002 | Induced sputum vs BAL for the diagnosis of PCP in patients with HIV | Cross-sectional | (1) Adults with HIV > 14 yrs (2) admitted with respiratory symptoms ≥ 7 days, ± radiological signs of pulmonary disease or (3) admitted with radiological changes without respiratory symptoms | (1) PCP prophylaxis received in 30d prior to admission (2) cachexia/deterioration (Karnofsky 20 - 30%), (3) decreased level of consciousness (4) Pa02 < 80mmHg on NP02 at 2L/min (5) FEV1 drop of 20% or decompensation during sputum induction (6) non-effective sputum induction (7) study consent refusal | Brazil (Rio Vermelho) | Inpatient | Induced sputum and/or BAL: GMS (Grocott-Gomori methenamine silver) | 60 | NS | Nil | 34.6 (SD ±7) | NS | 45 | 45 | NA |
| McLeod (1990)^[43]^  Nov 1986 - Sep 1987 | PCP in patients with AIDS in Central Africa | Prospective | Symptoms of pulmonary disease with/without CXR changes, admitted to two study hospitals during study period, HIV positive, consenting to study and willing to have bronchoscopy. | No additional exclusion criteria | Zimbabwe (Harare) | Inpatient | BAL or transbronchial biopsy, or autopsy lung biopsy (n = 2). Giemsa, toluidine blue, methanamine silver | 37; CXR findings reported in PCP group: n = 8 | NS | NS | PCP group: mean 30 yrs | NS | 8 | 8 | NA |
| Miller (1989)^[44]^  NS | Empiric treatment without bronchoscopy for PCP | Prospective | Group 1: clinical + CXR features typical of PCP. Group 2: atypical clinical or CXR features of PCP. Both group 1 and group 2 sent for bronchoscopy. | Not able to perform bronchoscopy (non-consent (1) and too ill (3)) | United Kingdom (London) | NS | IF on BAL | 7 | NS | NS | NS | Mean Pa02 9.5 (Range 5.2 - 12.2)(n = 24) | 7 | 7 | NA |
| Opravil (1994)^[45]^  Jan 1989 - Dec 1989 | Shortcomings of chest radiography in detecting PCP | Prospective (Apr to Dec 1989), and retrospective (chart review, Jan to Apr 1989) | All patients with HIV and first episode of PCP during study period | NS | Switzerland (Zurich) | NS | Sputum/induced sputum (n = 8) or BAL (n = 95), staining: Giemsa, Toluidine blue, or IFA | 93 | 50cells/mm3 (range 4 - 810) | NS | 35 (range 20 - 64yrs) | Pa02 < 75mmHg in 36/63 | 93 | 93 | NA |
| Rozaliyani (2020)^[46]^  Jul 2008 - Feb 2009 | Laboratory findings and clinical characteristics of PCP and PTB in patients with HIV in Indonesia | Cross-sectional | (1) ≥ 15 yrs (2) HIV -positive (3) symptoms/signs of pneumonia (including pulmonary infiltrates) (3) no prior CTX prophylaxis | NS | Indonesia (Jakarta) | Inpatient | Sputum induction; IFA staining | 55 | PCP: 20.13 ± 12.56 | Nil | 29.3 ± 5.6 | Median Pa02 PCP: 77.24 ± 12.79 | 8 | 8 | NA |
| Udwadia (2005) ^[47]^  2000 - 2003 | PCP in HIV-infected patients from Mumbai | Prospective study | (1) Adults with HIV (2) admitted for tertiary private care for pulmonary complaint (3) IFA-proven PCP | NS | India (Mumbai) | Inpatient | IFA or GMS staining of spontaneously expectorated sputum, induced sputum (n = 21/38) BAL (n = 15) or transbrochial biopsy (n = 3) | 38 | 137 cells/mm3 | NS | 40.1 (18 - 61 yrs) | Pa02 < 70mmHg: 19/38 | 38 | 38 | NA |
| Cohen (1984)^[48]^  NS; 3 yrs | Pulmonary complications of AIDS | Retrospective cohort | Retrospective review of 52 patients fulfilling CDC criteria for AIDS with pulmonary symptoms and undergoing bronchoscopy | NS | USA (New York) | NS | Methenamine-silver or giemsa | 52; data for non-PCP group incomplete - only PCP cases included (n = 34) | NS | NS | NS | NS | 34 | 34 | NA |
| Diero (2004) ^[49]^  June 1984 - Feb 2001 | Developing a prediction model for PCP based on clinical/radiological features | Retrospective cohort (online record review) | Patients with HIV, admitted during study period with respiratory symptoms and either (1) radiographic infiltrates or (2) clinical evidence pneumonia + undergoing bronchoscopy/lung biopsy | NS | USA (Indianapolis) | Inpatient | Bronchoscopy and BAL/transbronchial lung biopsy or transthoracic lung biopsy: Giemsa, modified acid fast, methenamine silver, gram stains. | 299 | PCP: mean 60 ± 90, non-PCP: 179 ± 347 | PCP: 78/111, non-PCP 134/188 | PCP: 40.7 ± 6.7, non-PCP: 42.3 ± 8.4 | Mean Pa02: PCP: 96 ± 58mmHg, non-PCP: 112 ± 75mmHg | 111 | 111 | NA |
| Rosen (1985)^[50]^  Dec 1981 - Dec 1983 | Diagnosis of pulmonary complications of the acquired immune deficiency syndrome | Prospective | Persistent cough, dyspnoea or abnormal CXR | NS | USA (New York) | NS | Bronchoscopy (BAL, brush biopsy, transbronchial biopsy) with microscopy (methenamine silver or Giemsa) | 48; CXR reported for n = 32 cases of PCP | NS | NS | 35 (range 24 - 49) | Pa02 < 80mmHg in 21/27 | 32 | 32 | NA |
| Siika (2006) ^[51]^  Aug 2001 - Jul 2002 | Aetiology of chronic cough in hospitalised adults with negative smears for MTB in Nairobi | Prospective | Admitted to study hospital (1) adults with HIV (2) cough ≥ 21day (3) abnormal CXR (4) three sputum samples negative for AFBs (5) not known with chronic lung disease (6) consenting | AFB positive on initial sputa, abnormal clotting parameters, too unwell to withstand bronchoscopy, declining consent | Kenya (Nairobi) | Inpatient | Sputum and BAL testing done for all patients. IFA microscopy staining. | 62 | 42/60 with CD4 < 200cells/mm3 | NA | 36.2±9.9 | NS | 22 | 22 | NA |
| Huang (1999)^[52]^  Nov 1988 - Feb 1994 (substudy of Pulmonary complications of HIV Infection study - PCHIS) | Whether an algorithm incorporating CXR and DLCO can predict PCP | Prospective cohort | Adults part of PCHIS follow up who developed: new or worsening respiratory symptoms (cough/SOB unexplained fever) - referred for PCP evaluation. | Data not available from hospital admission records | USA (San Francisco) | Inpatient and outpatient | Induced sputum or BAL: microscopy | 467 total. In non-PCP group (n = 382), definitive diagnosis not found in 165 (43%) - comparative arm excluded | 336 (201 - 530) | NS | NS | NS | 85 | 85 | NA |
| Logan (1995)^[53]^  May 1988 - Sep 1993 | Causes of acute lung disease in the immunocompromised host + diagnostic accuracy of CXR | Cross-sectional, unclear if retrospective | Acute lung disease (diagnosed: lung biopsy/autopsy/BAL/transbronchial biopsy/FNA) with CXR within 72 hours of admission. People with HIV or immunocompromised | NS | Canada (Vancouver) | NS | Included only cases in which a definite diagnosis was made; open lung biopsy, autopsy, BAL, transbronchial biopsy. Staining method not reported. | 149 | NS | NS | NS | NS | 21 | 21 | NA |
| Orlovic (2001)^[54]^  Jul 1997 - May 1999 | Clinical and radiological features in patients with HIV and with dual MTB and *P. jirovecii* infection | Retrospective study | Retrospective review of laboratory records to identify patients with HIV and hospitalized with pneumonia with both *P.jirovecii* cysts and MTB detected (AFB smear, culture or both) | Dual results on specimens > 5 days apart | South Africa (Johannesburg) | NS | IF staining | 39; CXR reported: n = 38 | 90 (range 12 - 428) | 2/39 | 30 | NS | 38 | 38 | NA |

NS - not specified, BP - bacterial pneumonia, CAP - Community acquired pneumonia, MTB - mycobacterial tuberculosis, PTB pulmonary tuberculosis, BAL - bronchoalveolar lavage, CXR - chest X-ray, PCP - pneumocystis pneumonia, AIDS - acquired immunodeficiency syndrome, CTX - cotrimoxazole (trimethoprim-sulfamethoxazole), IV - intravenous, MAI mycobacteriam avium intracellulare, MAC - mycobacterium avium complex, KS - Kaposi's sarcoma, CMV - cytomegalovirus, GMS - Gomori methenamine silver stain, P.jirovecii - *Pneumocystis jirovecii*, SD - Standard deviation, PCHIS - "Pulmonary Complications of HIV Infection Study (PCHIS)", DC - discharge, , LRTI - Lower respiratory tract infection, LAD - lymphadenopathy, Sp02 - Pulse oximeter oxygen saturation, Pa02 - Partial pressure of oxygen in arterial blood, Sa02 - Oxygenation saturation in arterial blood, TA - tracheal aspirate, yrs – years

**Table S2B. Study radiology definitions and approach to chest X-ray review**

| Author (year); STUDY PERIOD | Radiology: DEFINITIONS AND METHOD OF cxr REVIEW |
| --- | --- |
| Chave (1989) ^[5]^  Apr 1985 - Mar 1988 | Not specified |
| Garay (1989) ^[6]^  NS | Not specified |
| Marggrander (2021) ^[7]^  Sep 2017 - Apr 2019 | Blinded radiology review, noting the appearance and distribution of interstitial pathologies, consolidations, and pleural effusion. |
| Stover (1985) ^[8]^  NS, 4 yrs | Not specified |
| Batungwanayo (1994)^[9]^  Jan 1990 - Nov 1990 | Not specified |
| Hargreaves (2001)^[10]^  Nov 1997 - Jun 1998 | Blinded review by 2 clinicians; categorised according to the number of zones involved (out of six), and according to the likelihood of PTB as 1) normal; 2) abnormal, 3) consistent or not consistent with MTB. |
| Schlossbauer (2007)^[11]^  NS | Standard evaluation (1) pleural effusion (yes/no), pulmonary involvement - interstitial (categories: reticular, nodular, reticulonodular, peri-bronchial) OR (interstitial +) alveolar (ground glass, or focal consolidation), adenopathy (bi-hilar or mono-hilar), localisation (apical segments, perihilar, middle segments, basal segments) |
| Heron (1985)^[12]^  Aug 1983 - Oct 1984 | Blinded radiology review, method as per (Kerr, 1984). |
| Le Minor (2008) ^[13]^  Sep 2002 - Oct 2004 | Abnormalities, usually opacities, were summarized as localized and/or diffuse shadowing. Also noted: pleural effusion, mediastinal adenopathy, and cavitation. In case of discordance, the specialists were asked to review the chest x-rays and to reach a consensus classification |
| Malin (1995)^[14]^  May 1992 - May 1993 | Chest radiographs (blinded to micro result) were categorised into four groups: (i) diffuse or patchy shadowing in all lung zones; (ii) fine or coarse reticulonodular shadowing (fine indicating a nodular component <1 mm), (iii) perihilar distribution to shadowing with sparing of apices and bases; (iv) presence of hilar lymph node enlargement. |
| Mateyo (2014) ^[15]^  NS | Not specified |
| Pozniak (1986) ^[16]^  NS | Not specified |
| Selwyn (1998)^[17]^  March 1986 - July 1994 | Admission chest radiograph results as dictated by the attending radiologist recorded. No study specific definitions applied. |
| Suster (1986)^[18]^  Feb 1982 - Nov 1984 | All CXRs reviewed by one of the authors; PA or lateral CXR taken within 3 days of tissue sampling. Method not specified. |
| Amin (1997)^[19]^  Mar 1987 - Feb 1995 | Review by 2 radiologists, blinded to clinical details. Noted: anatomic location of the dominant area of consolidation, number of lobes/segments involved, consolidation: confluent or patchy, presence or absence of reticular or reticulonodular opacities, discrete nodules, lymphadenopathy, associated collapse/atelectasis, cavitation, bronchiectasis and bronchial wall thickening. Agreement was reached by the two radiologists on the radiographic abnormalities present in each patient. |
| Amorosa (1990)^[20]^  1984 - 1988 | Admission CXR reviewed by 2 experienced radiologists, categories: lobar consolidation ± pleural effusion, nodules, simple round air-space density, bilateral diffuse lung densities, bilateral, patchy localised lung densities with/without pleural effusion, effusion only. |
| Ansari (2002)^[21]^  Jul 1997 - Jun 1998 | CXR closest to date of death: systematically reviewed by investigator (TAK or STN) and the hospital radiologist. |
| Estrada Chacón (2002)^[22]^  Jan 2001 - Jun 2001 | Radiological images assessed for : inflammatory opacity, effusion opacity, nodular opacity, cavity, interstitial infiltrate and radiographs without pleuropulmonary (PP) abnormalities. Location of lesions and diagnosis at discharge. |
| Kibiki (2007)^[23]^  NS | Blinded review by radiologist, noting the following characteristics: nodules (miliary: <3 mm; micronodules: 3-6 mm; macronodules: 6 mm – 3 cm); infiltrates (alveolar, interstitial or mixed), cavities and their diameter and other abnormalities (pleural effusion or thickening, masses (> 3 cm), hilar and/or mediastinal adenopathy). |
| Weinberg (1993)^[24]^  June 1998 - May 1989 | Not specified |
| Baughman (1993)^[25]^  NS; 9 months | Blinded radiologist; CXR graded on a three-point scale ror presence of disease in the upper, middle, and lower zones; noted presence of cysts or pneumothorax or both. |
| Christe (2019) ^[4]^  2005 - 2012 | Two subspecialised radiologists, blinded to HIV status. Reported as: consolidation, reticulation, ground glass, nodules (solid, subsolid), cysts, signs of fibrosis (honeycombing), airways (cuffing, mucus plugging, bronchiectasis), and pleural alterations (thickening, effusion), central LAD, and parenchymal disease distribution |
| de la Paz Bermúdez (2020)^[26]^  Jan 1996 - Jan 2014 | Typical PCP radiology pattern defined as (1) fine, bilateral, symmetrical interstitial infiltrate, perihilar, basal, hilar-basal, or diffuse throughout the lung without adenopathy or pleural effusion or (2) consolidation of the air space (alveolar or interstitial alveolar infiltrate) or (3) Presence of small cysts or pneumatoceles. Atypical PCP pattern: (1) Nodules or masses with or without cavitation, (2) Adenopathy (3) Pleural effusion, (4) miliary infiltrate (5) Lobar affectation (6) Asymmetry of the lesions or unilateral infiltrates or infiltrates in upper lobes |
| Duflo (1986)^[27]^  June 1982 - Dec 1984 | Not specified |
| Edelstein (1990)^[28]^  June 1987 - Dec 1989 | Not specified |
| Engelberg (1984) ^[29]^  Mar 1981 - Oct 1982 | Not specified |
| Kaouech (2009) ^[30]^  Apr 2005 - Dec 2007 | Systematic CXR review with noting of changes (1 category per patient): interstitial, alveolar-interstitial, alveolar, nodular or consolidation. Distribution - diffuse or localised. |
| Leach (1991)^[31]^  NS | Unclear if blinded. CXR only described as normal, focally abnormal or diffusely abnormal. |
| Mones (1986)^[32]^  1981 - 1984 | Blinded review. Four grades of severity were defined - 1 grade pr pt: 0 (normal roentgenogram), 1 (mild), 2 (moderate), and 3 (severe). Grade 1: interstitial finely reticular or reticulonodular infiltrates distributed in a uniform manner throughout both lungs. In grade 2, in addition to the interstitial changes: patchy areas of alveolar consolidation. In grade 3: confluent areas of alveolar consolidation (appearance of ARDS). Unusual roentgenographic findings noted: pleural effusions, cavitation of the parenchymal infiltrates, and hilar-mediastinal LAD. |
| Peruzzi (1991)^[33]^  Jan 1985 - Mar 1989 | As per Brenner et al |
| Wang (2005)^[34]^  Jan 1992 - Oct 2004 | Not specified |
| Wollschlager (1984)^[35]^  1981 - Jul 1983 | Not specified |
| Brenner (1987)^[36]^  Jan 1983 - Apr 1986 | Blinded radiology review. Patterns evaluated: nodular, interstitial, or alveolar, distribution, and symmetry of lung infiltrates. Interstitial infiltrates graded on a scale of 1 to 4: Grade 1, subtle increased perihilar interstitial markings; Grade 2, interstitial markings more prominent and extending laterally without evidence of alveolar infiltrates; Grade 3, gross interstitial disease with mild alveolar infiltrates; Grade 4, extensive interstitial and alveolar infiltrates. |
| Choi (2003)^[37]^  1996 - 2002 | Not specified. |
| DeLorenzo (1987)^[38]^  1981- 1985 | Systematic review for: interstitial pattern (granular, nodular, reticular, or reticulonodular appearance), alveolar: air-space filling, the presence of air bronchograms, or confluent acinar infiltrate, or interstitial-alveolar: characteristics of both interstitial and alveolar categories. These three may be associated with (1) Honeycomb: presence of air-containing cystic spaces up to 1em in diameter and separated by a coarse reticular network or (2) Pulmonary cyst: thin-walled air-containing space 1 cm or greater in diameter, and/or hilar enlargement |
| Ewig (1996)^[39]^  May 1989 - June 1995 | The distribution of the infiltrates reported; with patterns of parenchymal infiltrates were graded as either: absent (0); reticular or reticular-nodular (1); ground-glass (2); or airspace filling (3). A severity score was calculated by multiplying the type of the parenchymal infiltrate and the number of areas affected. A presentation of PCP was considered as "atypical" if one of the following patterns was present: predominant or isolated apical infiltrates; presence of cysts and/or cavitations; pneumothorax; extrapulmonary or disseminated infection. |
| Griffiths (1995)^[40]^  NS | Two patterns of abnormality - reticular nodular (interstitial), and a fine perihilar generalised loss of radiolucency with loss of the normal lung markings (alveolar). |
| Mane (2015)^[41]^  NS | Not specified |
| SILVA (2007) ^[42]^  Jan 2001 - Dec 2002 | Categories (1 per patient): interstitial, alveolar, interstitial-alveolar, alveolar + mediastinal adenomegaly, interstitial + pneumothorax, normal. |
| McLeod (1990)^[43]^  Nov 1986 - Sep 1987 | Not specified |
| Miller (1989)^[44]^  NS | Categorised as typical or atypical for PCP: bilateral alveolar or interstitial shadowing or normal. Atypical CXR for PCP: focal abnormalities such as lobar consolidation or pleural effusion(s) or mediastinal lymphadenopathy |
| Opravil (1994)^[45]^  Jan 1989 - Dec 1989 | CXRs reviewed by radiologists independently, blinded (some health controls added to the group without radiologists knowing which films came from controls), and given a score distinguishing between none, interstitial and acinar infiltrates |
| Rozaliyani (2020)^[46]^  Jul 2008 - Feb 2009 | Not specified |
| Udwadia (2005) ^[47]^  2000 - 2003 | Not specified |
| Cohen (1984)^[48]^  NS; 3 yrs | Not specified |
| Diero (2004) ^[49]^  June 1984 - Feb 2001 | Not specified |
| Rosen (1985)^[50]^  Dec 1981 - Dec 1983 | Not specified |
| Siika (2006) ^[51]^  Aug 2001 - Jul 2002 | CXR reported on by 2 radiologists blinded to clinical data. Grouped into: (1) diffuse or patchy lung shadowing, all lung zones (2) fine or coarse reticulonodular shadowing (fine = nodular component < 1mm), (3) perihilar shadowing, sparing of apices and bases (4) hilar LAD (5) multiple abnormalities |
| Huang (1999)^[52]^  Nov 1988 - Feb 1994 (substudy of Pulmonary complications of HIV Infection study - PCHIS) | Standardized review by study physician: parenchymal abnormality if present - interstitial, alveolar, and nodular infiltrate[s], nodule[s], and cavities]) and their location (right/left lung, upper/middle/lower lung zones), and number of zones involved. |
| Logan (1995)^[53]^  May 1988 - Sep 1993 | Two independent blinded observers. Noted: presence of parenchymal opacification, nodules, linear opacities, bullae or cystic changes, lymphadenopathy, and pleural effusions. Parenchymal opacification was defined as a homogeneous increase in opacity and ranged from mild opacification without obscuration of underlying markings to dense consolidation. The nodules were assessed for number, size, and presence of cavitation. The distribution of the predominant abnormality was classified as affecting the upper, middle, or lower lung zones. |
| Orlovic (2001)^[54]^  Jul 1997 - May 1999 | Not specified |

CXR – chest X-ray, LAD – lymphadenopathy, OR – odd’s ratio, PA – posterior anterior, PCP – Pneumocystis pneumonia, PTB – pulmonary TB

| Author (year) | Amin (1997) | Amorosa (1990) | Ansari (2002) | Batungwanayo (1994) | Baughman (1993) | Brenner (1987) | Chave (1989) | Choi (2003) | Christe (2019) | Cohen (1984) | de la Paz Bermúdez (2020) | DeLorenzo (1987) | Diero (2004) | Duflo (1986) | Edelstein (1990) | Engelberg (1984) | Estrada Chacón (2002) | Ewig (1996) | Garay (1989) | Griffiths (1995) | Hargreaves (2001) | Heron (1985) | Huang (1999) | Kaouech (2009) | Kibiki (2007) |
| --- | --- | --- | --- | --- | --- | --- | --- | --- | --- | --- | --- | --- | --- | --- | --- | --- | --- | --- | --- | --- | --- | --- | --- | --- | --- |
| Study design and sampling frame (../2) | 1 | 1 | 2 | 2 | 2 | 2 | 1 | 1 | 1 | 1 | 1 | 1 | 1 | 1 | 1 | 1 | 1 | 2 | 1 | 1 | 2 | 1 | 2 | 1 | 2 |
| Sample size calculation (../2) | 1 | 1 | 1 | 1 | 1 | 1 | 1 | 1 | 1 | 1 | 1 | 1 | 1 | 1 | 1 | 1 | 1 | 1 | 1 | 1 | 1 | 1 | 2 | 1 | 1 |
| Selection of participants (../3) | 2 | 2 | 2 | 2 | 2 | 3 | 1 | 2 | 2 | 1 | 2 | 2 | 2 | 2 | 3 | 2 | 1 | 3 | 1 | 1 | 2 | 1 | 1 | 1 | 3 |
| Diagnosis and case definitions (../2 | 2 | 2 | 2 | 1 | 1 | 2 | 1 | 1 | 2 | 1 | 2 | 2 | 1 | 1 | 1 | 1 | 0 | 2 | 1 | 1 | 2 | 1 | 3 | 1 | 2 |
| Prevalence denominator (../2) | 2 | 2 | 2 | 2 | 2 | 2 | 2 | 2 | 2 | 2 | 2 | 2 | 2 | 2 | 2 | 2 | 2 | 2 | 2 | 2 | 2 | 2 | 2 | 2 | 2 |
| Prevalence numerator (../2) | 2 | 2 | 2 | 2 | 2 | 2 | 1 | 2 | 1 | 1 | 1 | 2 | 2 | 2 | 2 | 1 | 2 | 2 | 2 | 2 | 2 | 1 | 2 | 2 | 2 |
| Total score | 10 | 10 | 11 | 10 | 10 | 12 | 7 | 9 | 9 | 7 | 9 | 10 | 9 | 9 | 10 | 8 | 7 | 12 | 8 | 8 | 11 | 7 | 12 | 8 | 12 |
| Attrition bias | NA | L | NA | NA | NA | L | NA | NA | NA | L | NA | L | NA | L | L | L | NA | L | L | L | L | NA | H | U | L |
| Selection bias | L | U | U | U | U | U | H | U | U | U | U | U | U | H | U | U | H | U | U | H | H | H | U | H | U |

Table S3. Quality of, and risk of bias in, 51 included studies.

L – Low risk (study appears to be free of bias), H – High risk (there is at least one important risk of bias that could alter the CXR features reported), U – Unclear risk (there may be a risk of bias, but there is either (1) insufficient information to assess whether an important risk of bias exists or (2) insufficient rationale or evidence that an identified problem will introduce bias), NA – not applicable (cross sectional analysis/no follow up period)

| Author (year) | Leach (1991) | Le Minor (2008) | Logan (1995) | Malin (1995) | Mane (2015) | Marggrander (2021) | Mateyo (2014) | McLeod (1990) | Miller (1989) | Mones (1986) | Opravil (1994) | Orlovic (2001) | Peruzzi (1991) | Pozniak (1986) | Rosen (1985) | Rozaliyani (2020) | Schlossbauer (2007) | Selwyn (1998) | Siika (2006) | Silva (2007) | Stover (1985) | Suster (1986) | Udwadia (2005) | Wang (2005) | Weinberg (1993) | Wollschlager (1984) |
| --- | --- | --- | --- | --- | --- | --- | --- | --- | --- | --- | --- | --- | --- | --- | --- | --- | --- | --- | --- | --- | --- | --- | --- | --- | --- | --- |
| Study design and sampling frame (../2) | 2 | 1 | 1 | 2 | 2 | 2 | 2 | 2 | 2 | 1 | 2 | 1 | 1 | 1 | 1 | 1 | 1 | 1 | 2 | 2 | 1 | 1 | 2 | 1 | 1 | 1 |
| Sample size calculation (../2) | 1 | 1 | 1 | 1 | 1 | 1 | 1 | 1 | 1 | 1 | 1 | 1 | 1 | 1 | 1 | 1 | 1 | 2 | 1 | 1 | 1 | 1 | 1 | 1 | 1 | 1 |
| Selection of participants (../3) | 3 | 2 | 2 | 3 | 2 | 2 | 2 | 2 | 3 | 2 | 2 | 1 | 3 | 2 | 2 | 2 | 2 | 3 | 3 | 3 | 2 | 2 | 2 | 2 | 2 | 2 |
| Diagnosis and case definitions (../2 | 1 | 2 | 1 | 2 | 1 | 1 | 1 | 1 | 1 | 2 | 2 | 1 | 2 | 0 | 1 | 1 | 2 | 2 | 2 | 2 | 1 | 1 | 1 | 1 | 1 | 1 |
| Prevalence denominator (../2) | 2 | 2 | 1 | 2 | 2 | 2 | 2 | 2 | 2 | 2 | 2 | 2 | 2 | 2 | 2 | 2 | 2 | 2 | 2 | 2 | 2 | 2 | 2 | 2 | 2 | 2 |
| Prevalence numerator (../2) | 2 | 2 | 1 | 2 | 2 | 2 | 2 | 1 | 2 | 2 | 2 | 2 | 2 | 2 | 1 | 1 | 2 | 2 | 2 | 2 | 1 | 2 | 2 | 1 | 2 | 1 |
| Total score | 11 | 10 | 7 | 12 | 10 | 10 | 10 | 9 | 11 | 10 | 11 | 8 | 11 | 8 | 8 | 8 | 10 | 12 | 12 | 12 | 8 | 9 | 10 | 8 | 9 | 8 |
| Attrition bias | L | NA | NA | L | L | H | NA | NA | NA | NA | L | NA | L | NA | NA | NA | NA | NA | L | NA | L | L | L | L | L | L |
| Selection bias | L | H | H | U | U | U | U | U | U | U | L | H | U | H | H | H | U | L | U | L | H | U | U | U | U | H |

L – Low risk (study appears to be free of bias), H – High risk (there is at least one important risk of bias that could alter the CXR features reported), U – Unclear risk (there may be a risk of bias, but there is either (1) insufficient information to assess whether an important risk of bias exists or (2) insufficient rationale or evidence that an identified problem will introduce bias), NA – not applicable (cross sectional analysis/no follow up period)

**Figure S1. Prevalence of nodular (A) and alveolar infiltrates (B) and consolidation (C) in adults with HIV-associated PCP.**


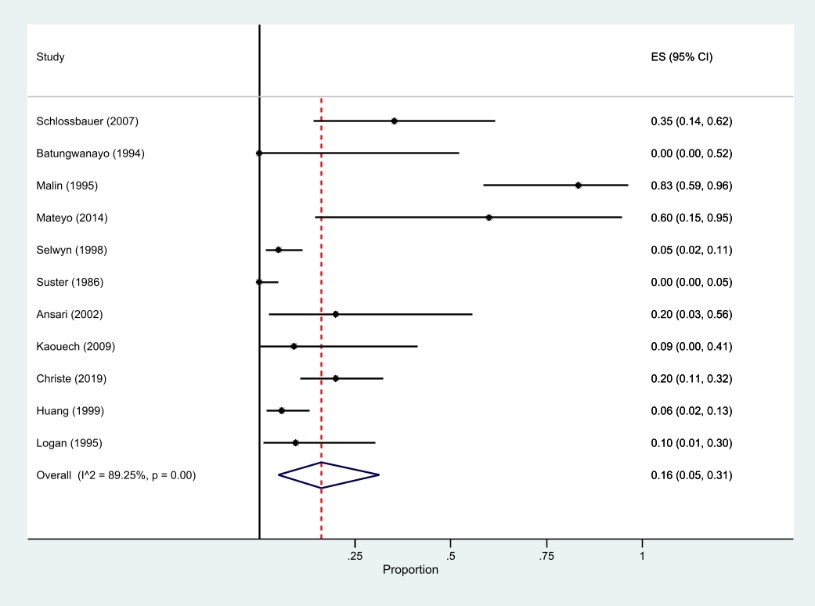


**A**

**
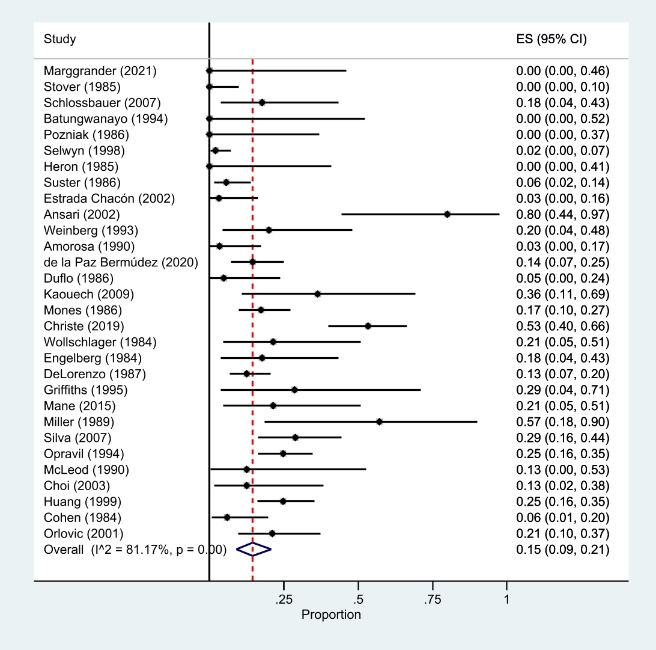
**


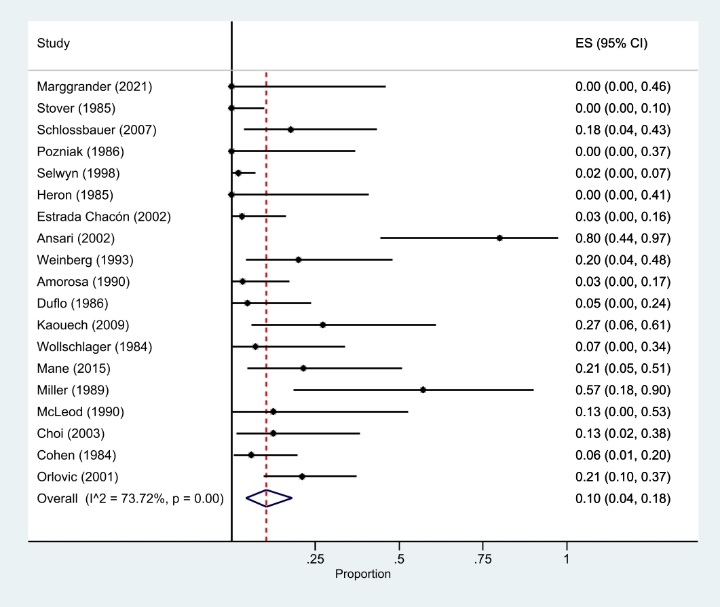


**C**

**B**

**Figure S2. Prevalence of cystic changes (A), pleural effusion (B), central lymphadenopathy (C), cavitation (D) and pneumothorax (E) in in adults with HIV-associated PCP.**


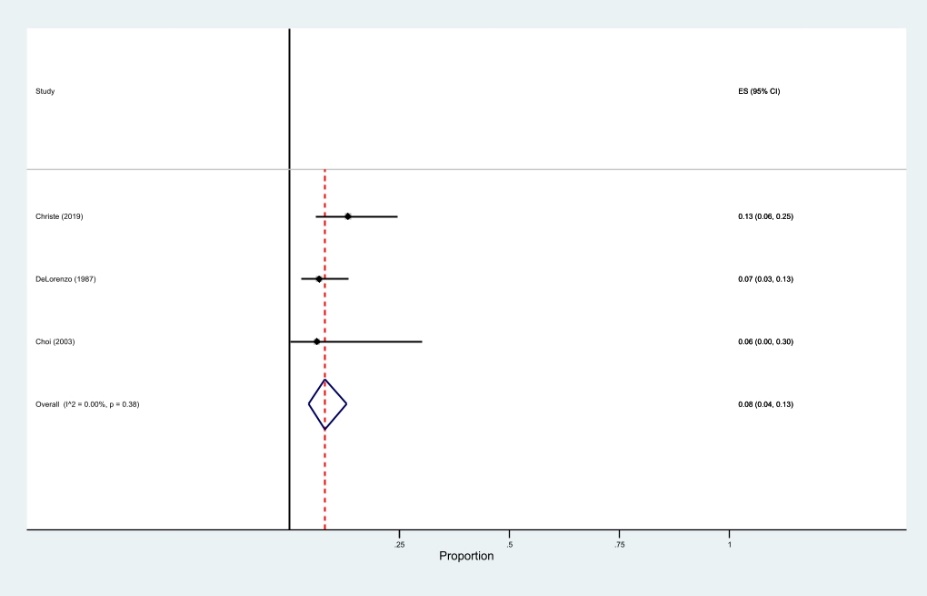


**A**


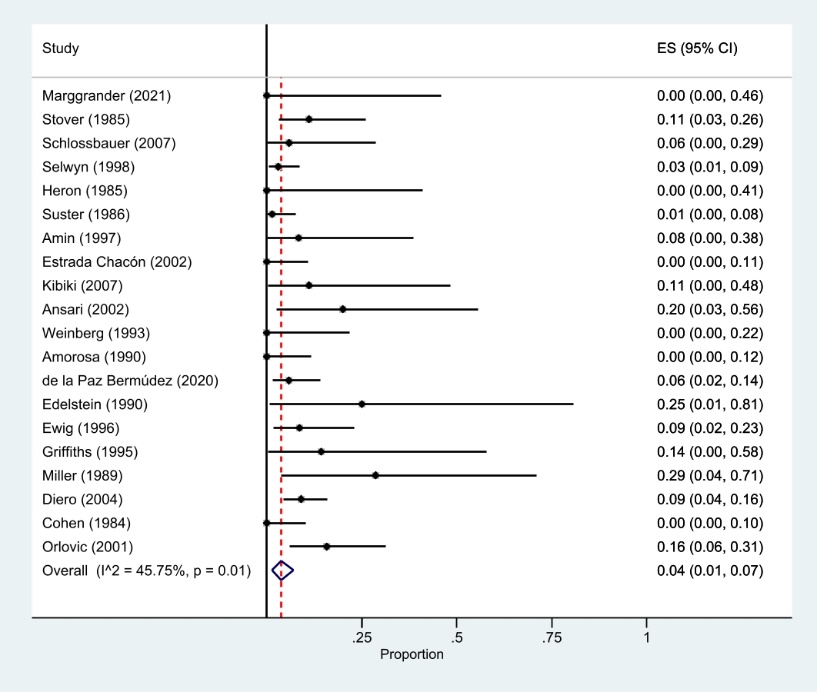


**B**


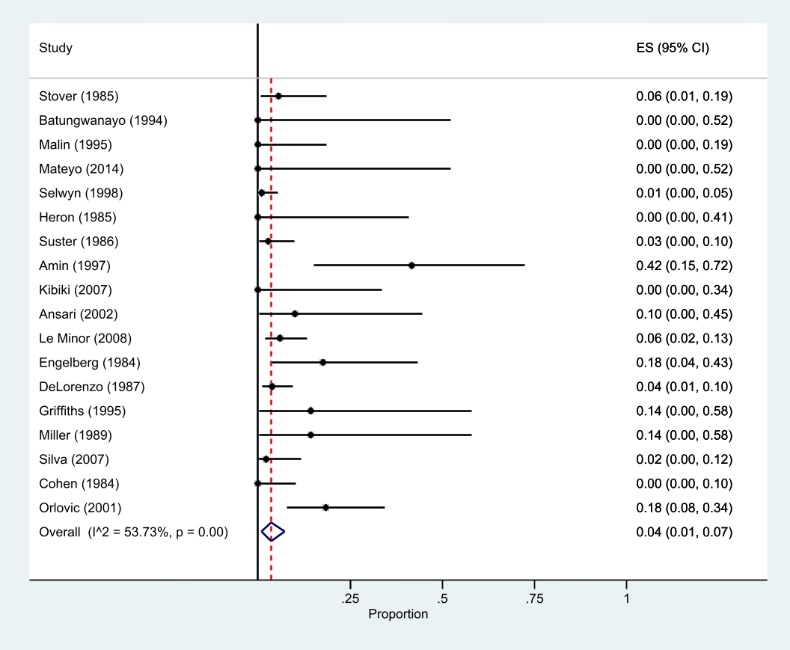


**C**


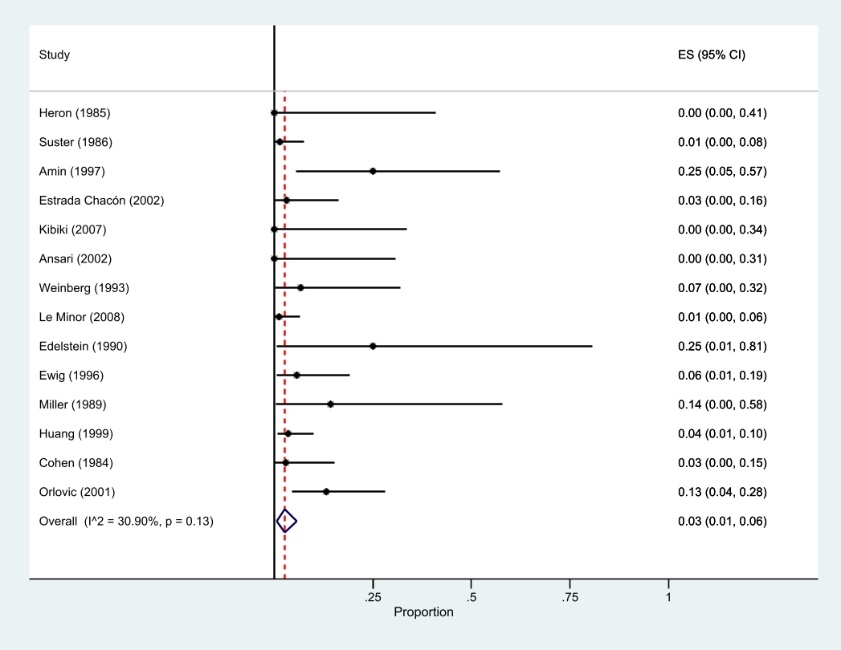


**D**


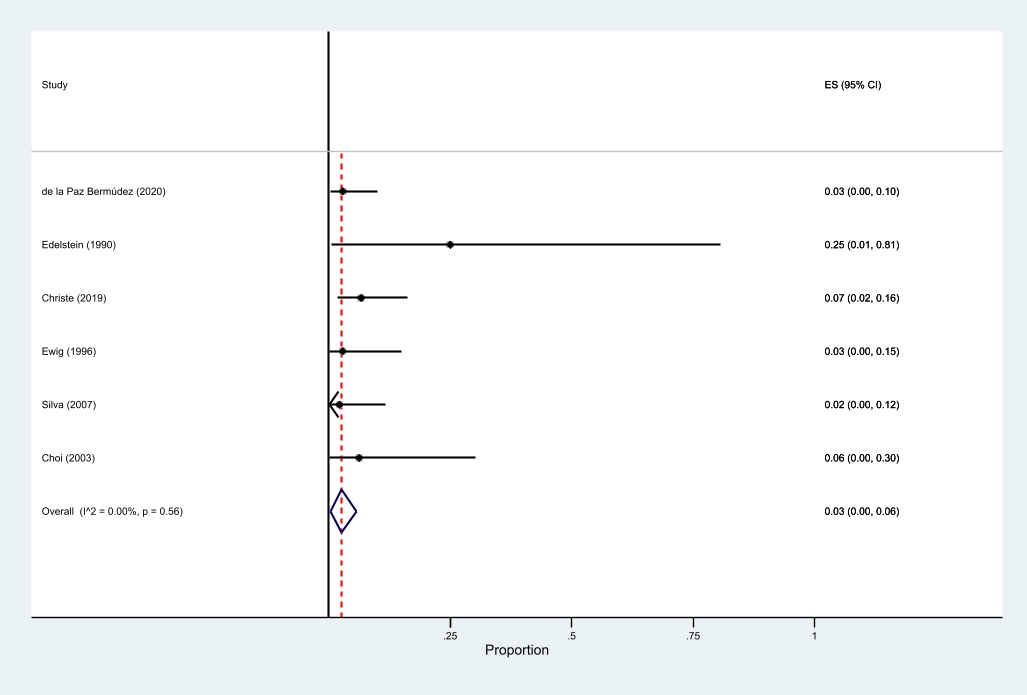


**E**

**Figure S3. Prevalence of interstitial-alveolar disease (A) and upper zone disease (B) in adults with HIV-associated PCP, stratified by studies conducting a systematic versus unspecified radiology review.**


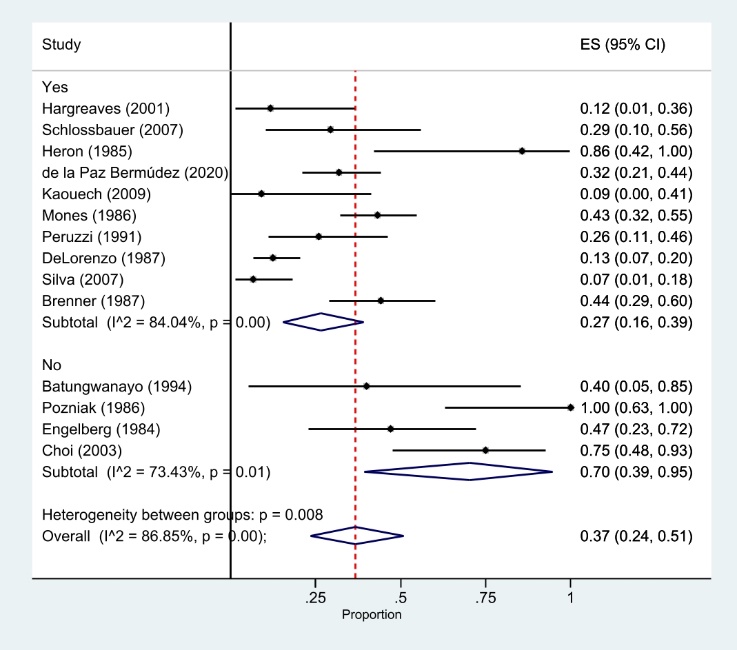


**A**


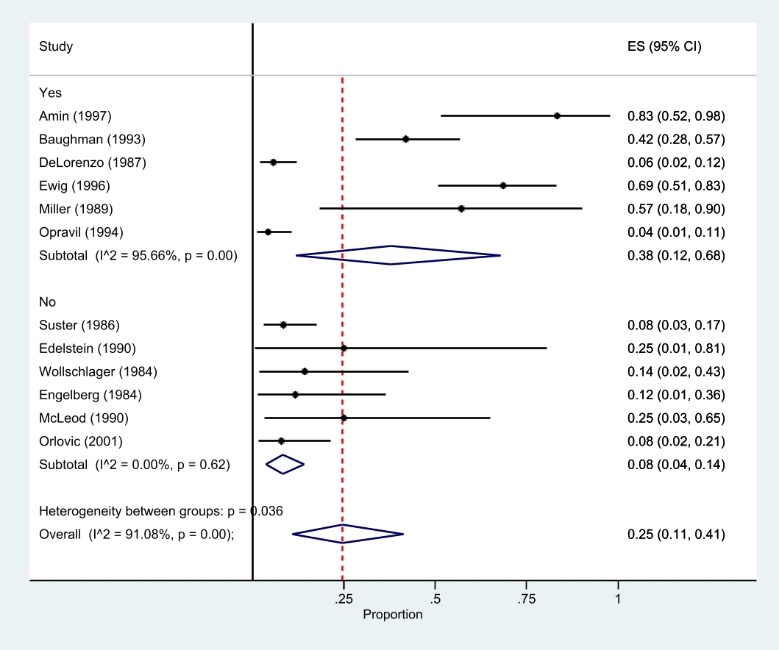


**B**

**Figure S4. Prevalence of miliary infiltrate (A), alveolar infiltrate (B), consolidation (C) and pleural effusion (D) in in adults with HIV-associated PCP, stratified by studies reporting radiological features in exclusive PCP cases.**


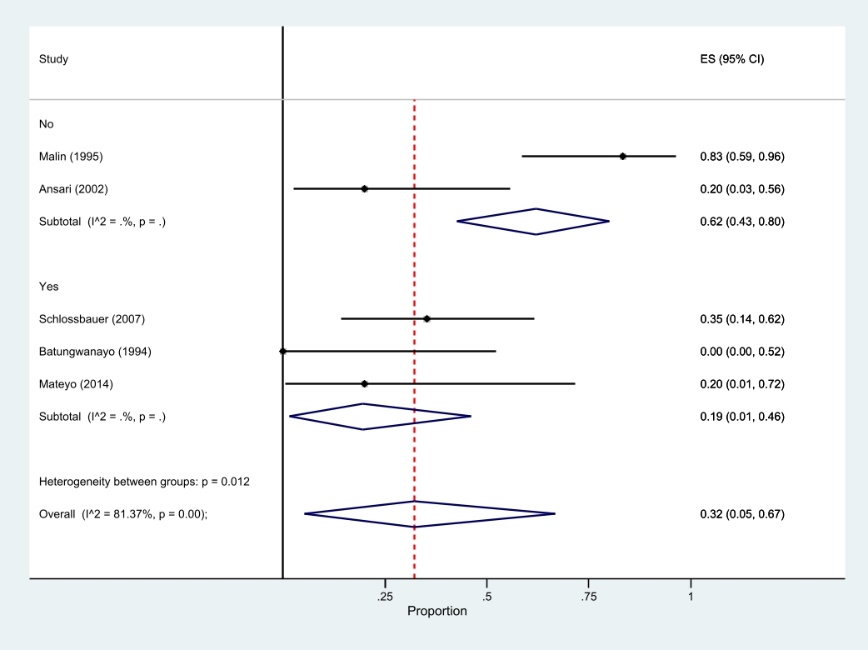


**A**


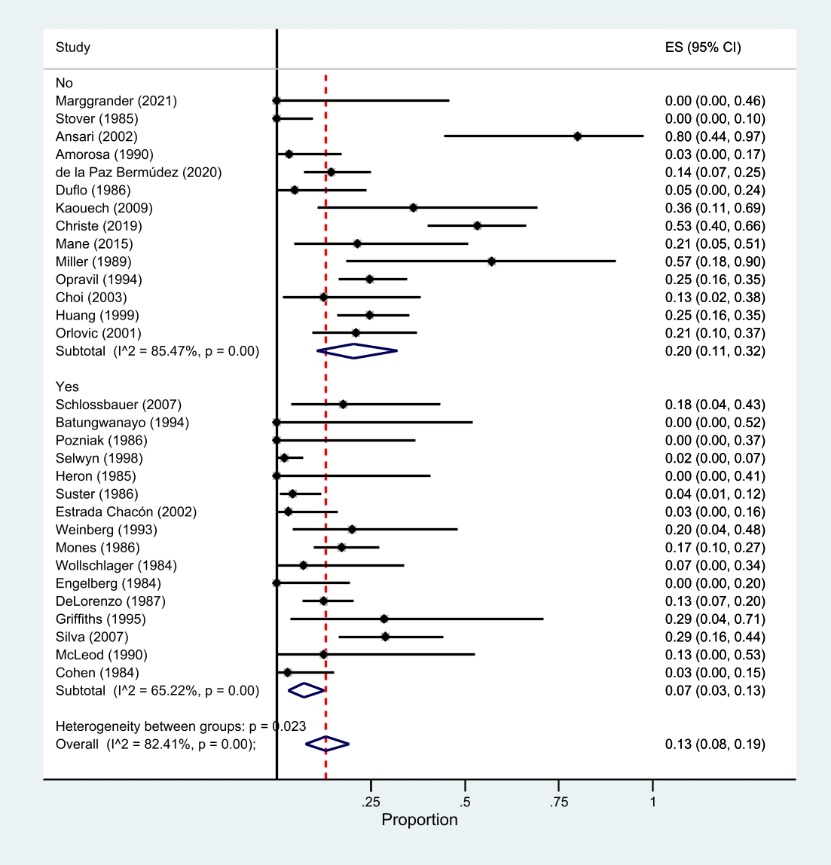


**B**


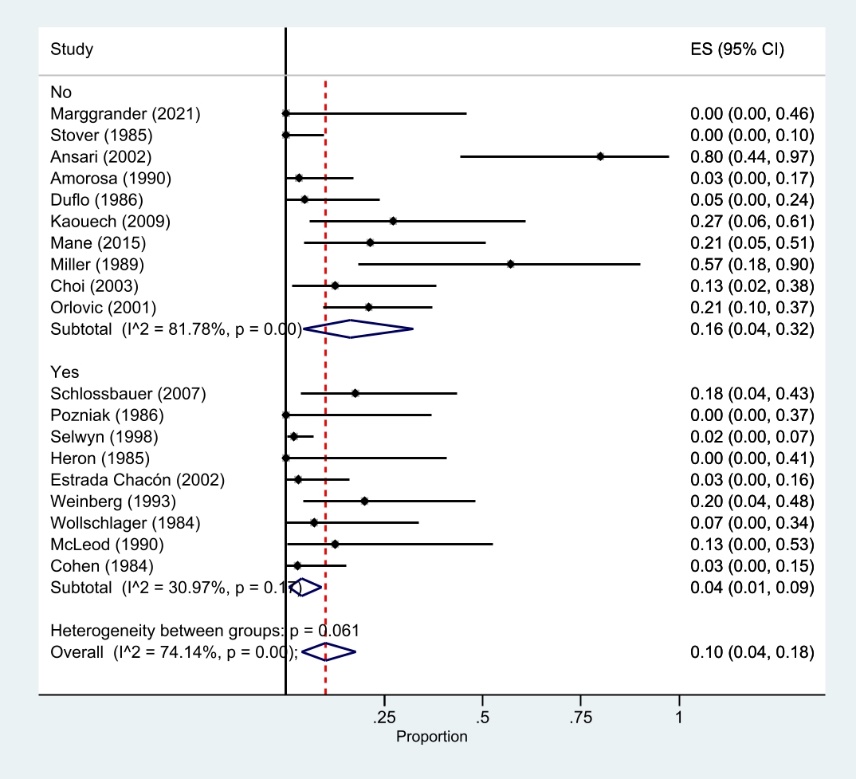


**C**


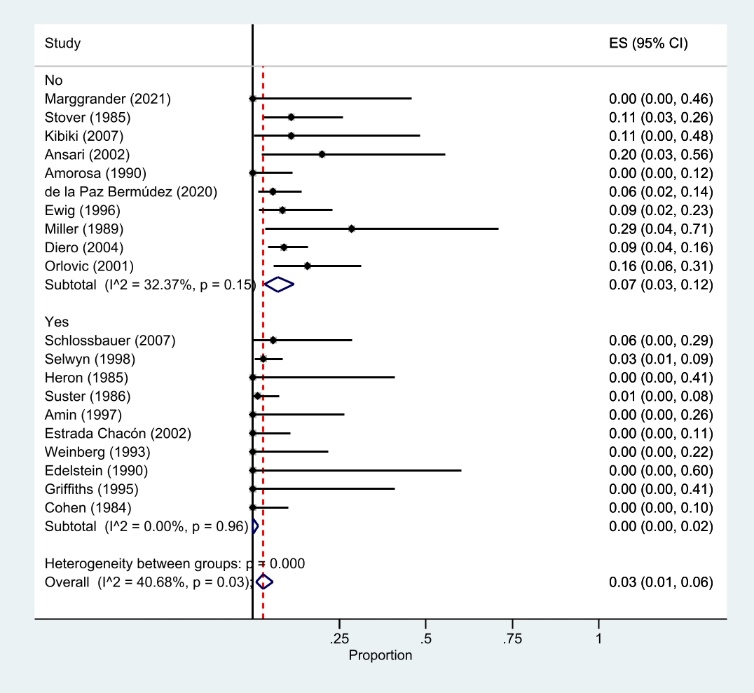


**D**

**Figure S5.** **Prevalence of diffuse CXR changes in adults with PCP, stratified by median study CD4 count.**

**
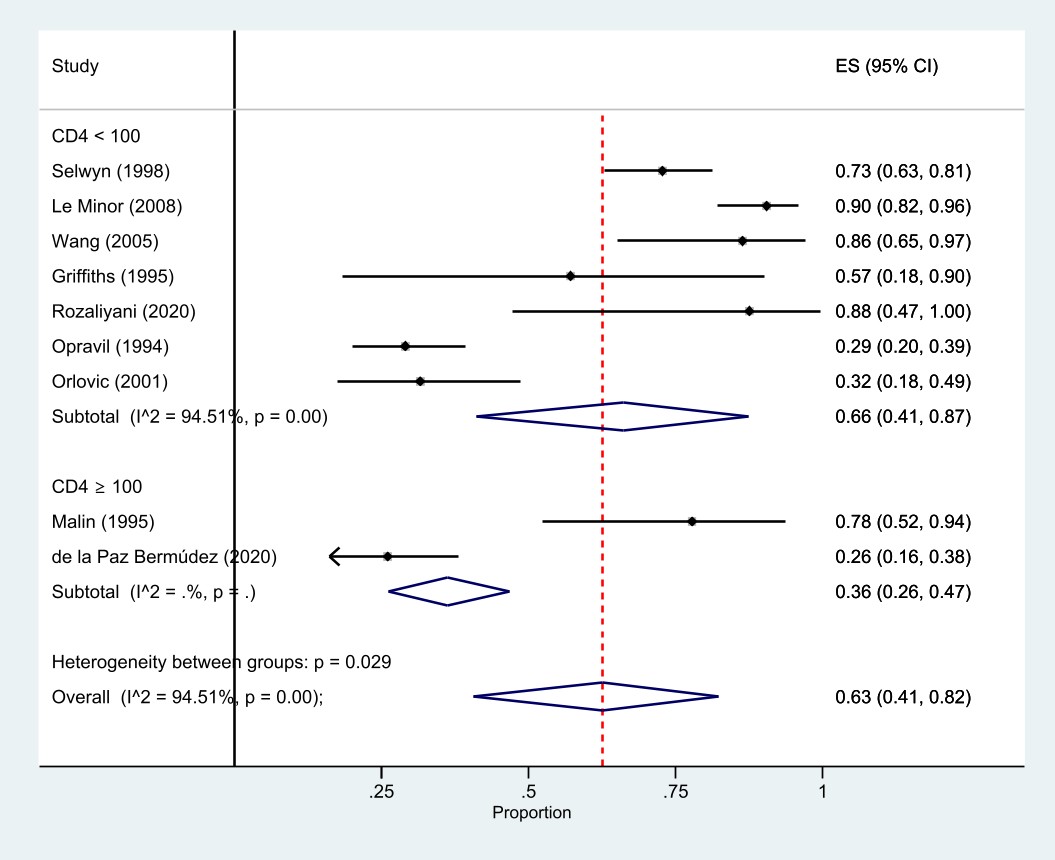
**

**Figure S6.** **Prevalence of interstitial-alveolar infiltrate (A), consolidation (B), pleural effusion (C), diffuse CXR changes (D) and focal CXR changes (E) in adults with PCP, stratified by African versus non-African study setting.**


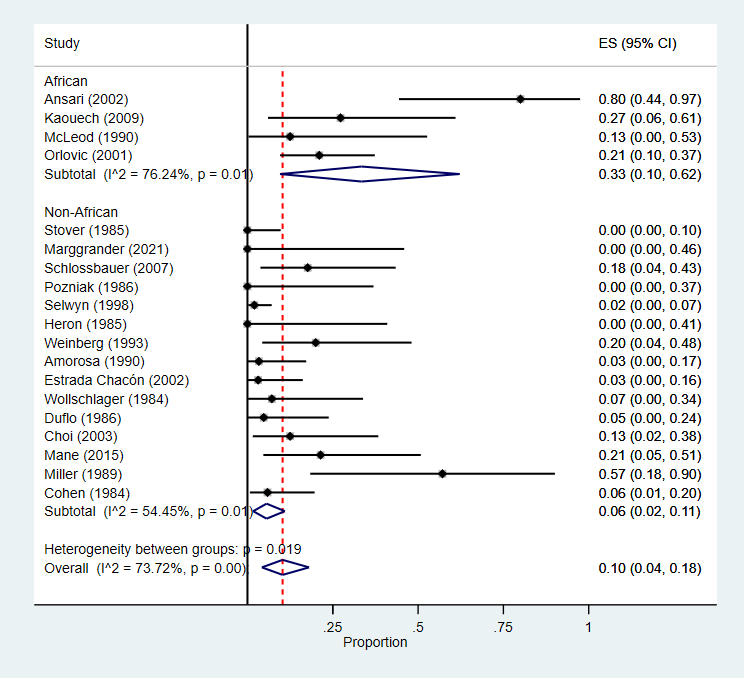


**B**


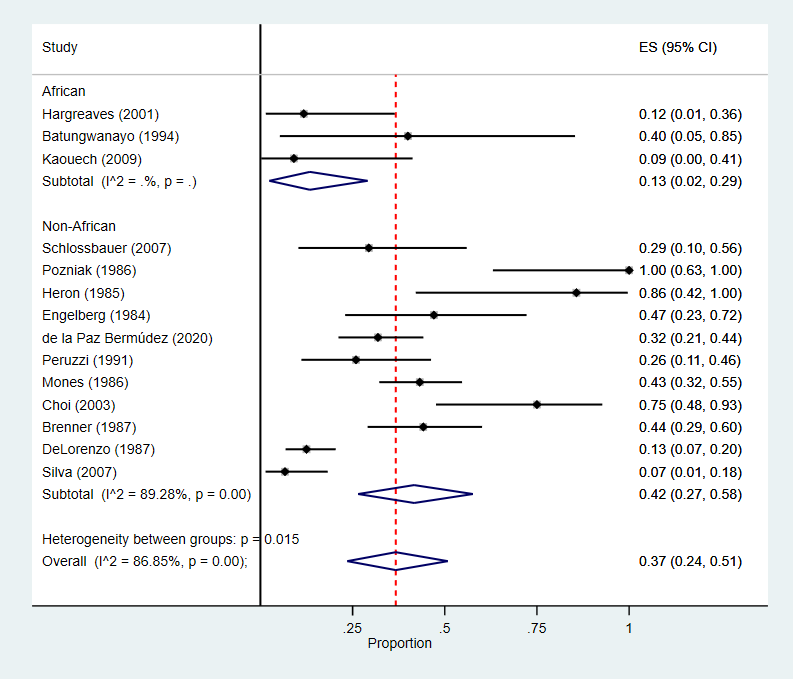


**A**


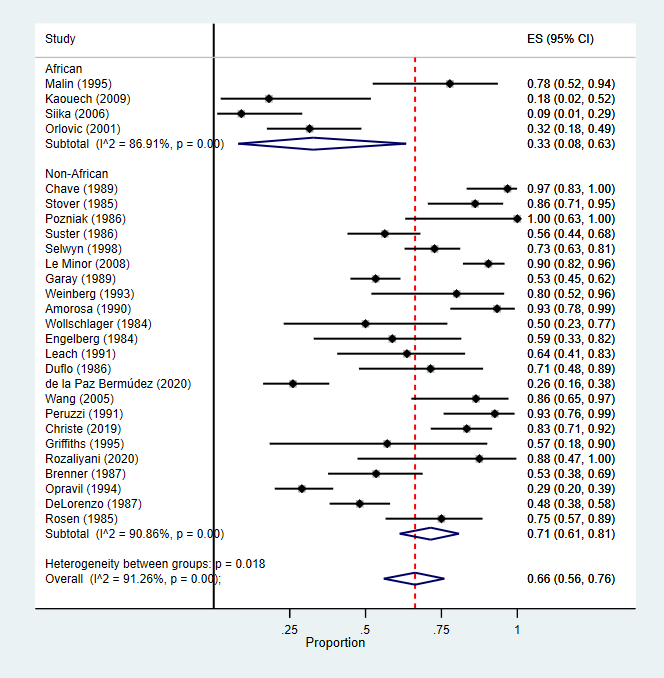


**D**


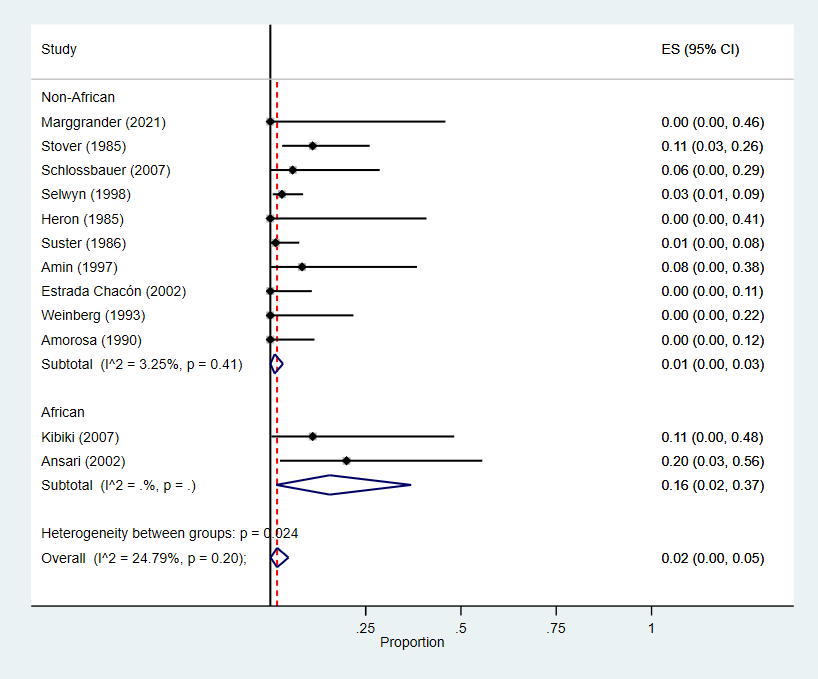


**C**


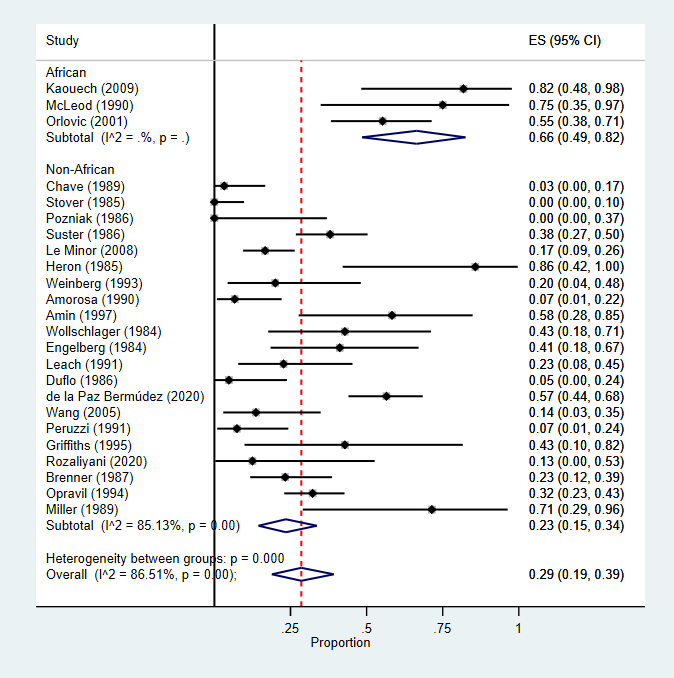


**E**

Differences between African and non-African studies in all displayed features lost significance when restricting analysis to studies enrolling exclusive PCP cases only.

**Figure S7. CXR features predictive of PCP versus non-PCP respiratory disease in adults with HIV: interstitial infiltrate (A), interstitial-alveolar infiltrate (B), diffuse CXR changes (C), and any infiltrate (D)**


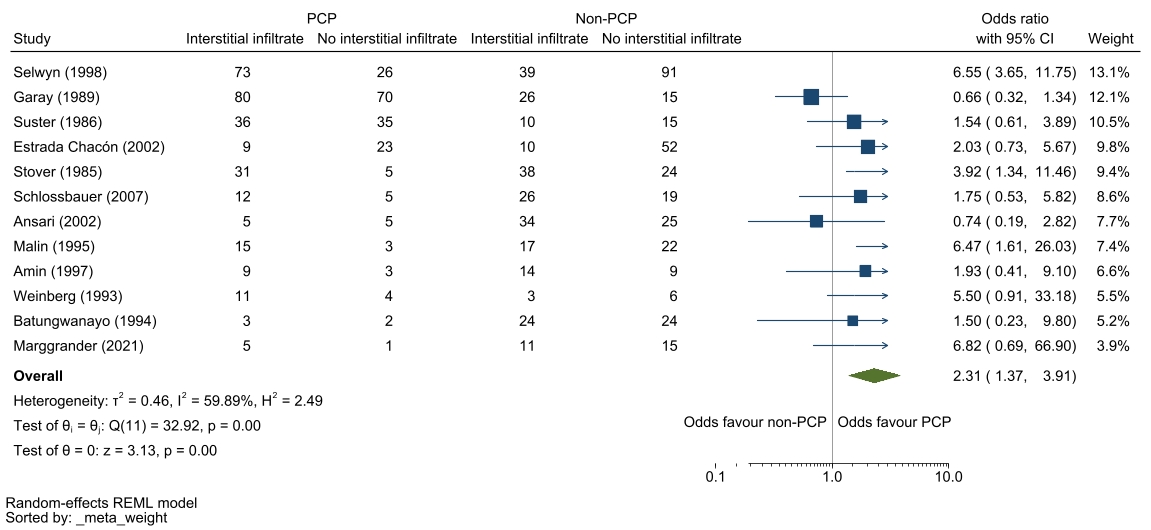


A


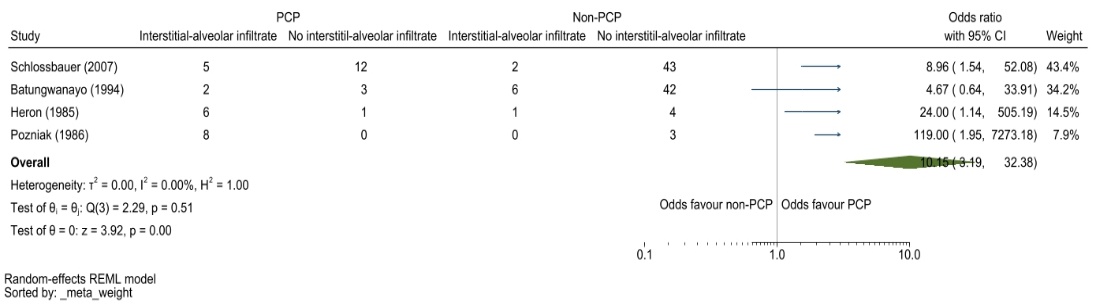


B


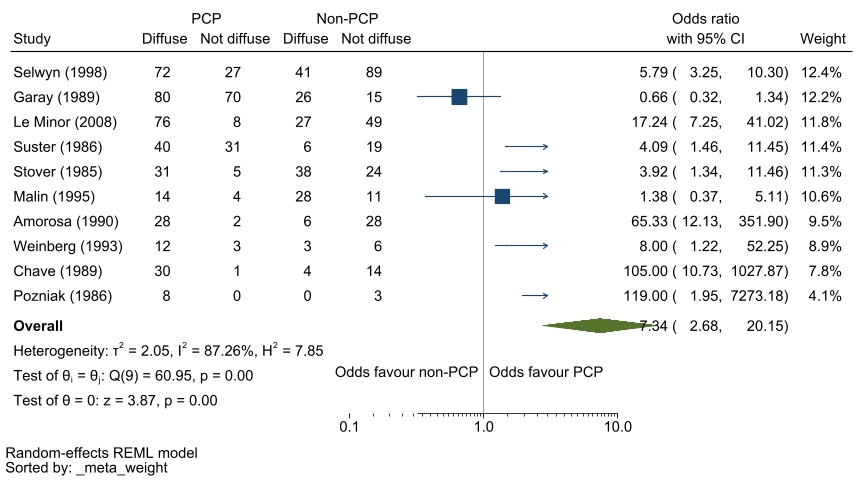


C


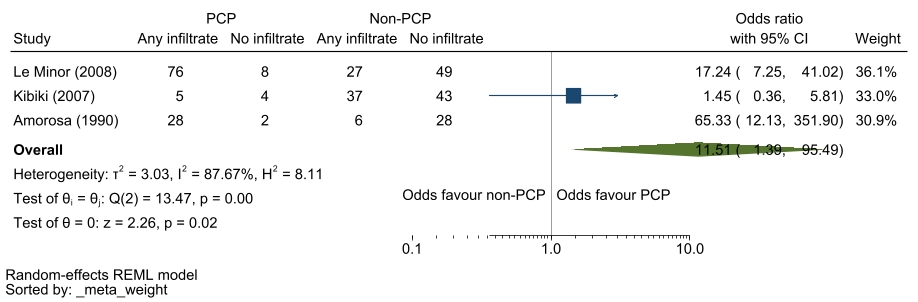


D

**Figure S8. CXR features that correlate with non-PCP respiratory disease in adults with HIV: alveolar infiltrate (A), consolidation (B), pleural effusion (C), central lymphadenopathy (D), focal CXR changes (E).**


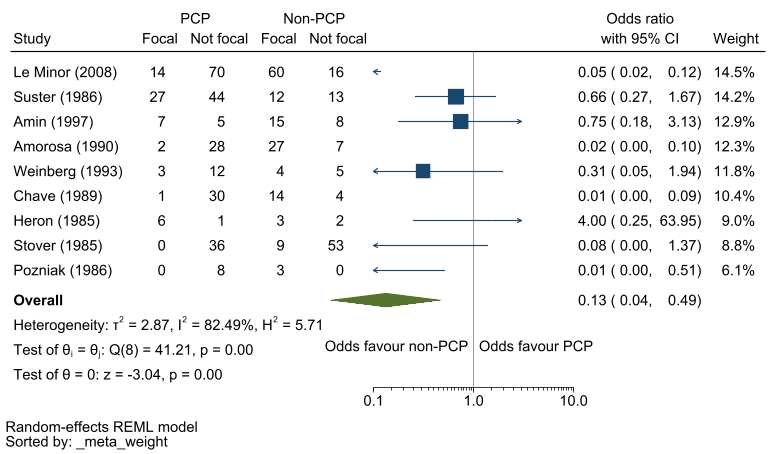


C


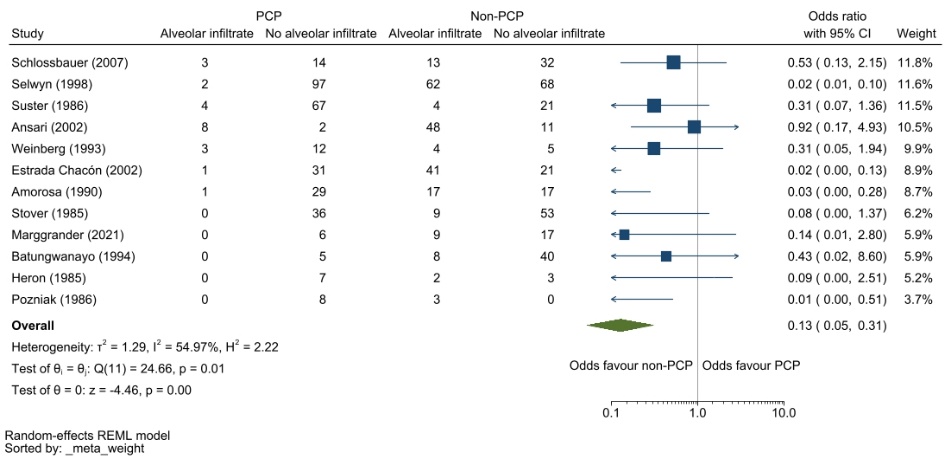


A


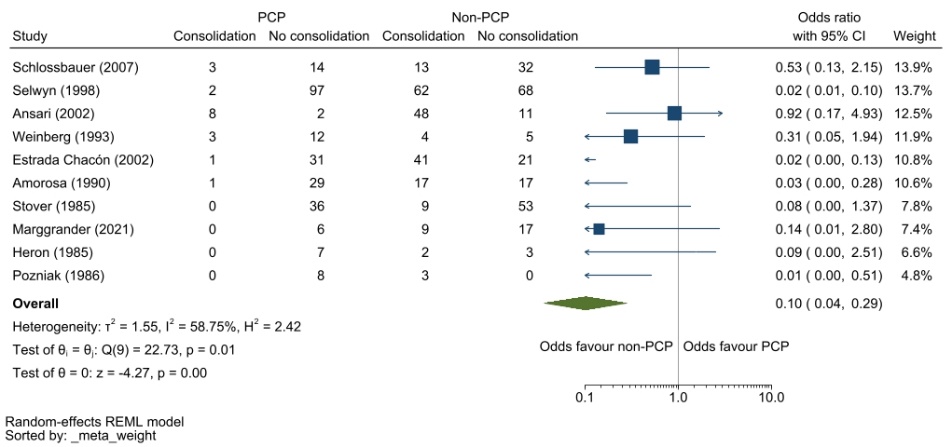


B


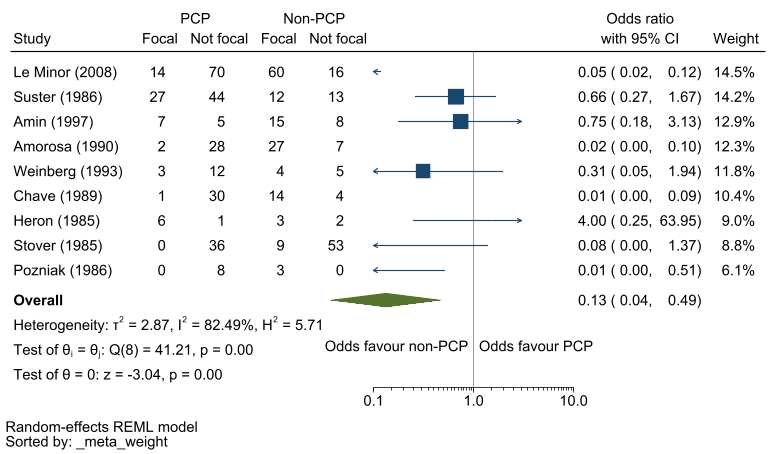


C


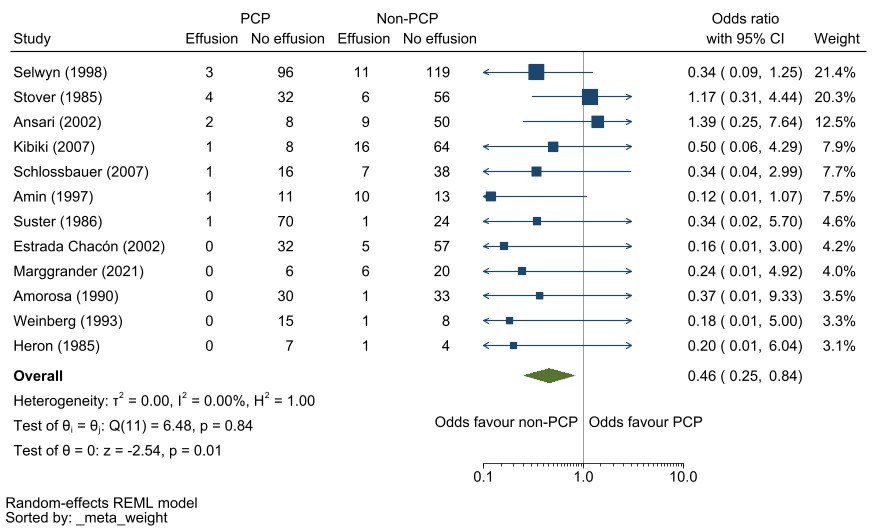


D


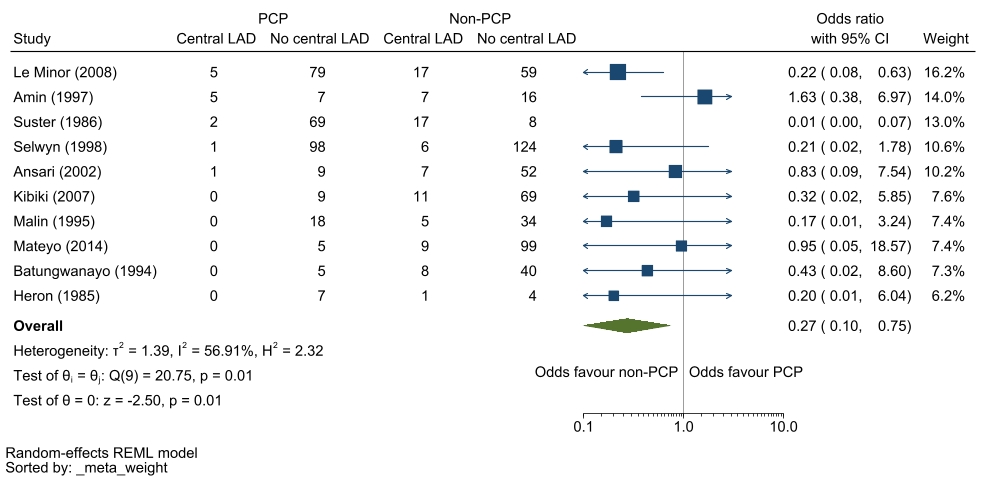


E

**Figure S9. Odds of PCP versus non-PCP respiratory disease in adults with HIV with cavitation on CXR.**

**
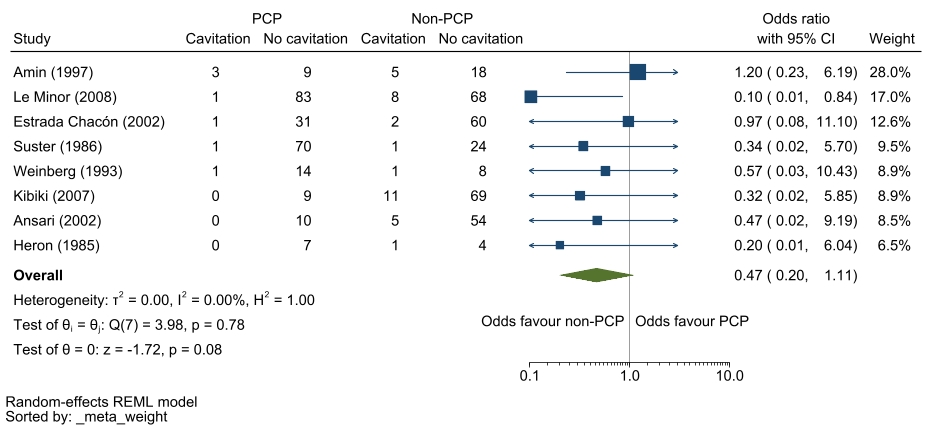
**

**Figure S10. Odds of PCP versus non-PCP respiratory disease in adults with HIV with a normal CXR (A), interstitial-nodular infiltrate (B) or miliary infiltrate (C).**


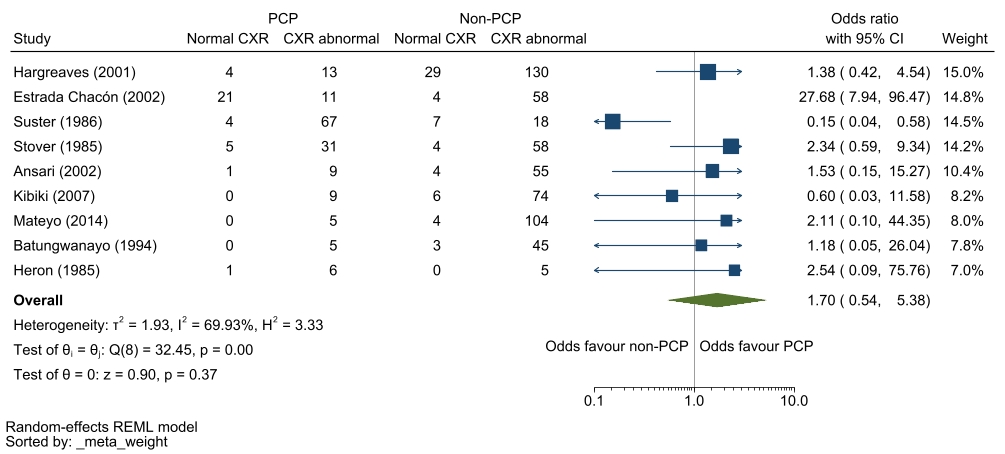


A


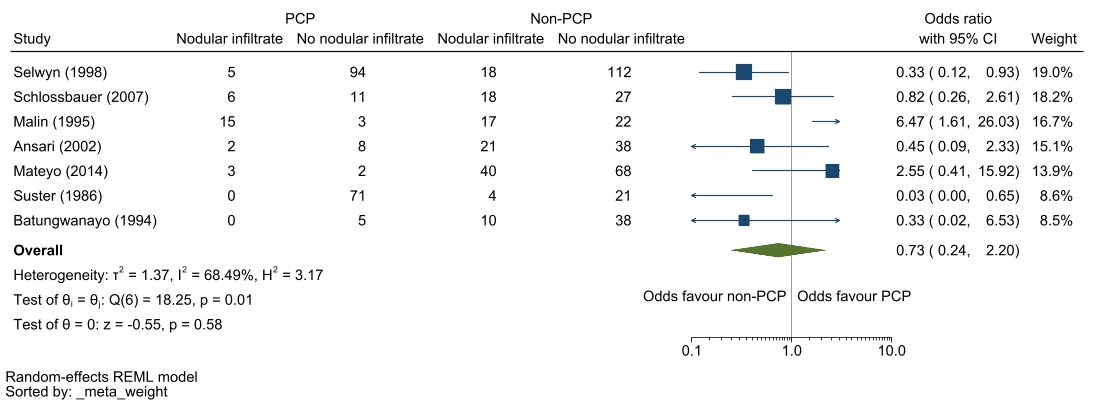


B


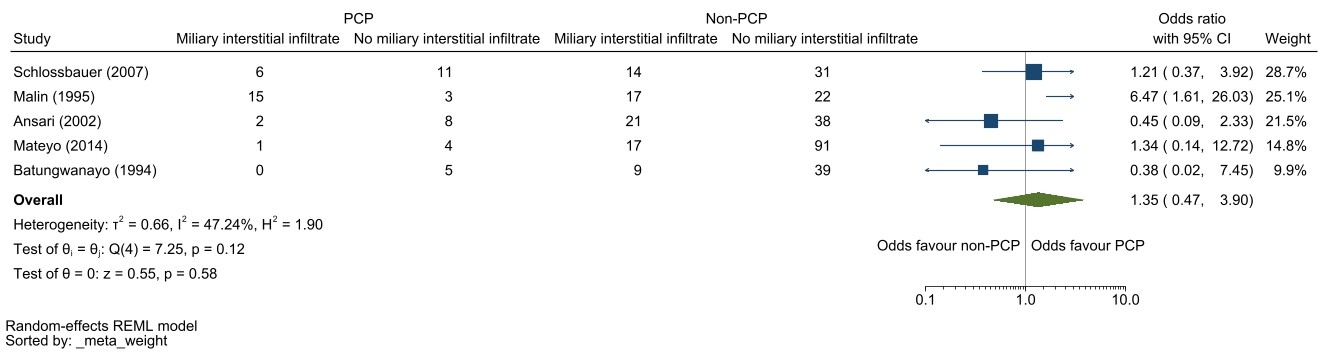


C

**Figure S11. Odds of PCP versus non-PCP respiratory disease in adults with HIV with (A) interstitial infiltrate, stratified by median study CD4 count (B) interstitial-alveolar infiltrate, stratified by systematic versus unspecified CXR review, (C) effusion, stratified by systematic versus unspecified CXR review, (D) central lymphadenopathy, stratified by time-period of enrolment, and (E) focal CXR changes, stratified by systematic versus unspecified CXR review.**

Interaction on subgroup analysis for (A) interstitial infiltrate: in 2 studies (n = 119) with median CD4 ≥ 100, OR 3.2 (0.9 – 11.4), difference between groups: p = 0.52; (B) interstitial-alveolar infiltrate: in 2 studies (n = 64) where radiological review method not specified, OR 14.0 (95% CI 0.7 – 282.2), difference between groups: p = 0.91; (C) pleural effusion: in 4 studies (n = 447) where radiological review method not specified, OR 0.5 (95% CI 0.2 – 1.3), difference between groups: p = 0.63; (D) central lymphadenopathy: in 6 studies (n = 422) conducting systematic radiology review, OR 0.4 (95% CI 0.2 – 1.2), and in 4 studies (n = 491) where radiological review method not specified, OR 0.1 (95% CI 0.0 – 1.0), difference between groups: p = 0.31; (E) focal CXR changes: in 4 studies (n = 271) conducting a systematic radiology review, 0R 0.20 (95% CI 0.0 – 1.9) Difference between groups: p = 0.64


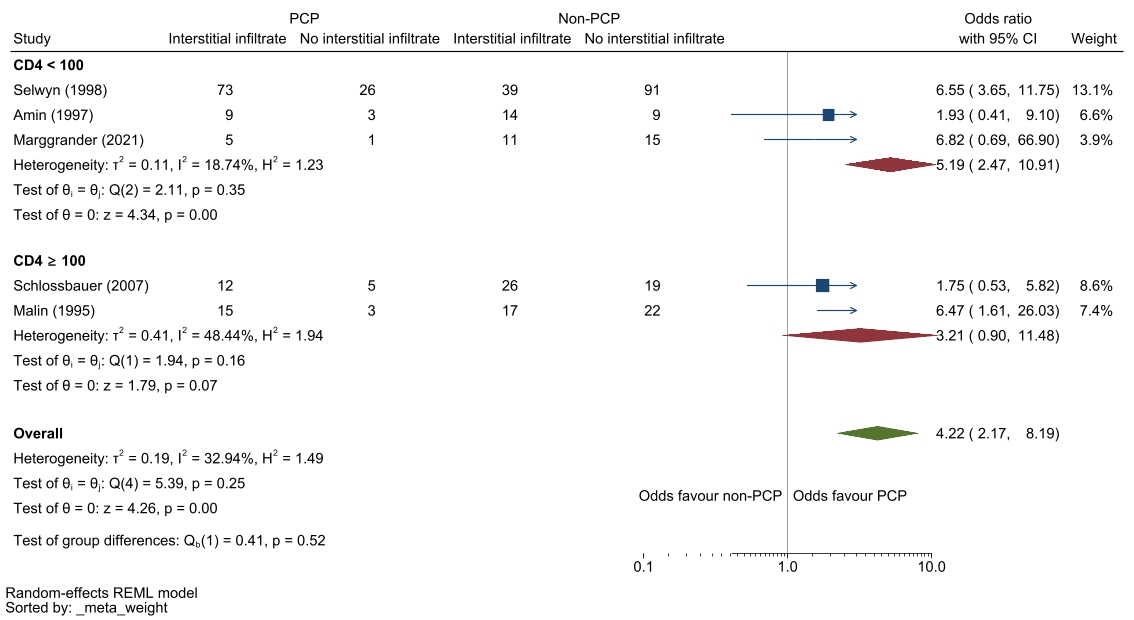


A


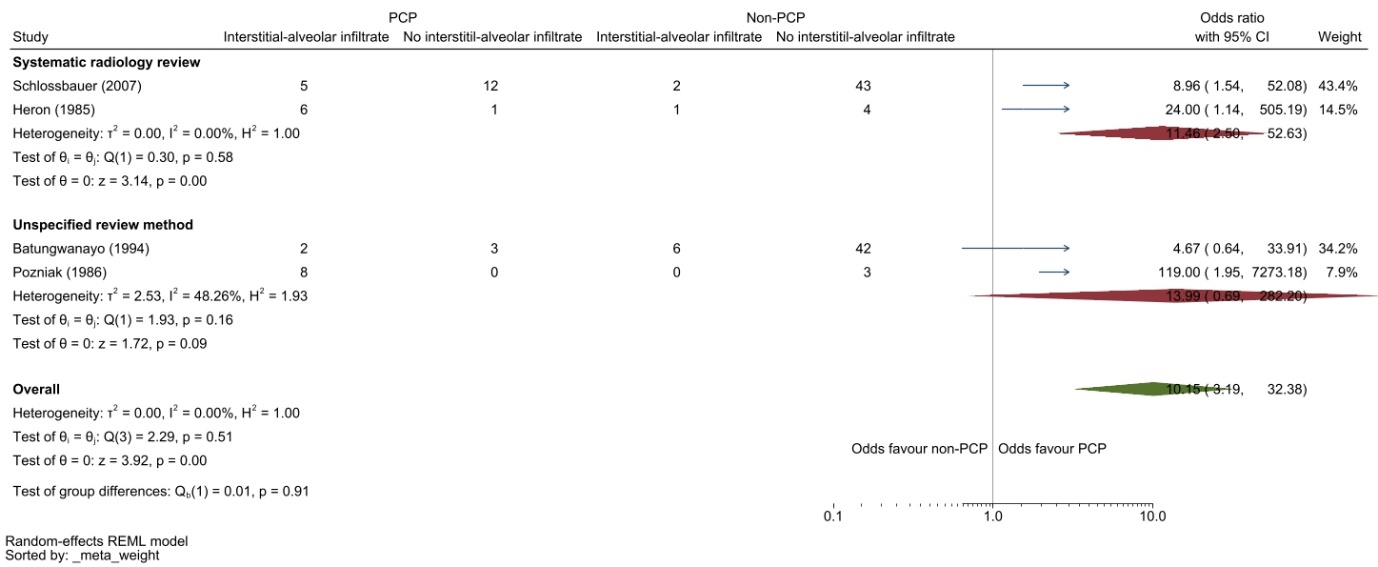


B


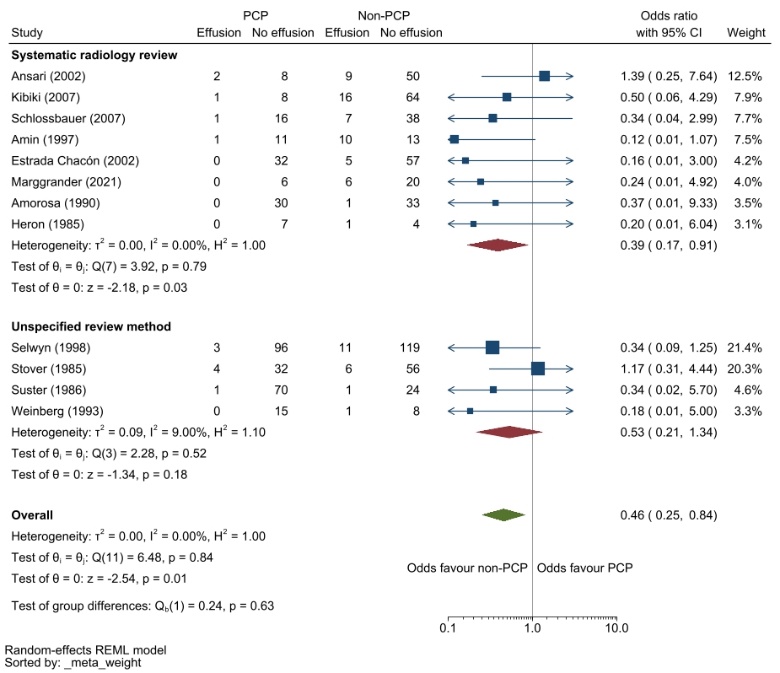


C


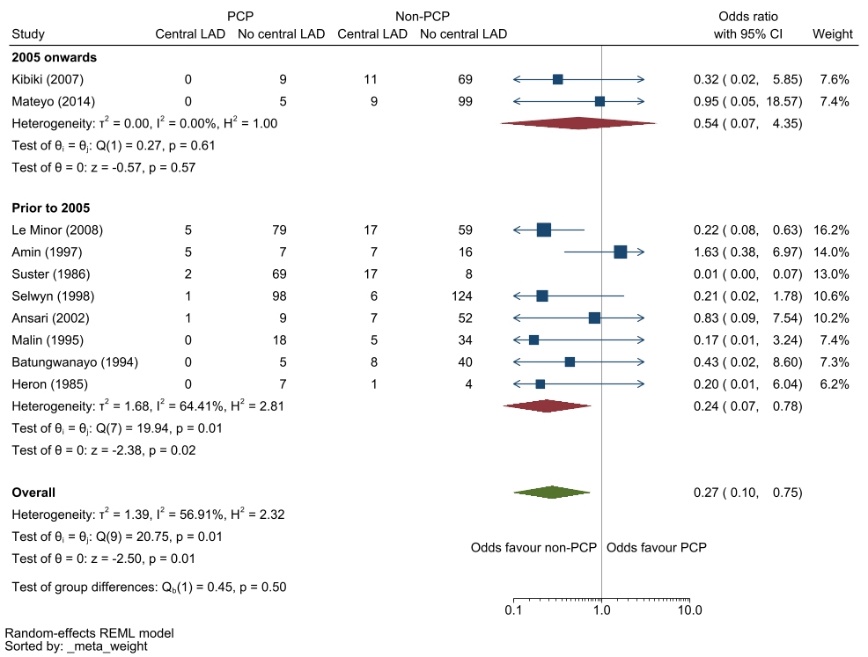


D


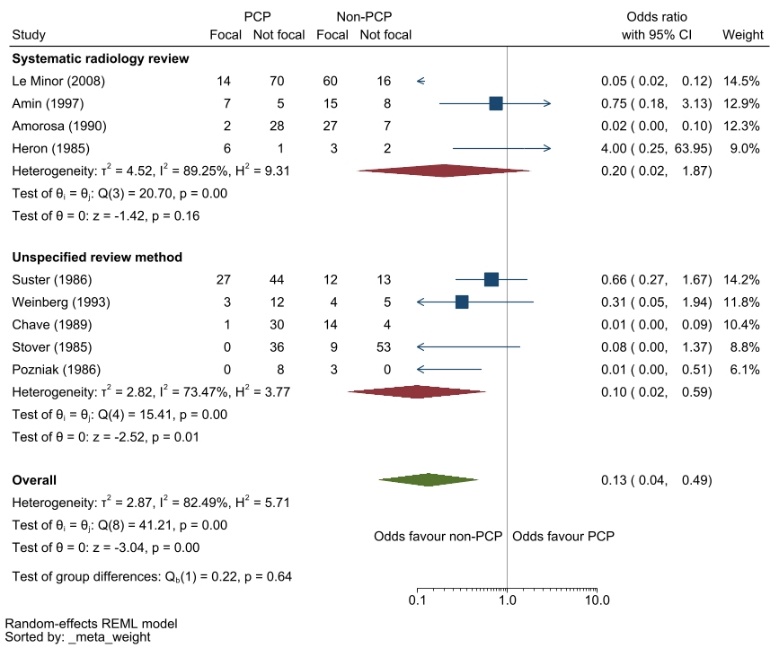


E

**Figure S12. Odds of PCP versus non-PCP respiratory disease in adults with HIV with (A) alveolar infiltrate and (B) pleural effusion, stratified by African versus non-African study setting.**


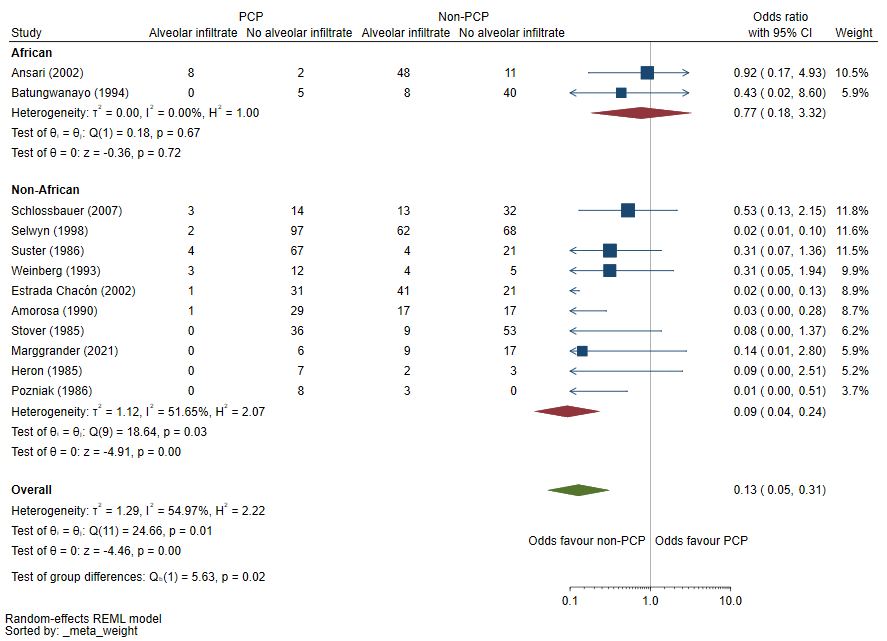


A


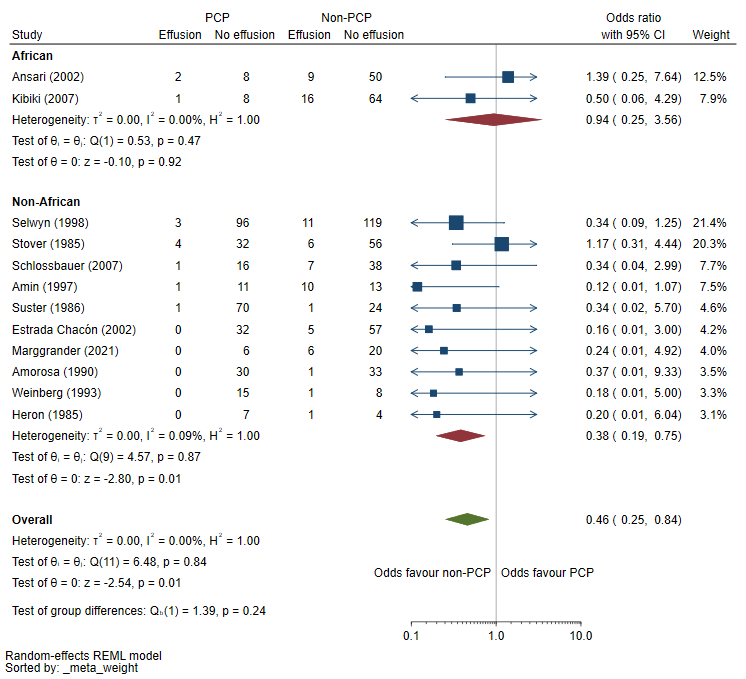


B

**Appendix 1. Search terms used on PubMed and adapted for use on other databases**

| #23 | Search: ((((((("x ray" OR x-ray OR xray OR radiography OR radiograph OR film OR imaging OR image OR X-ray OR "X ray" OR Xray)) AND ((chest OR thorax OR pulmonary))) OR ((CXR OR cxr))) OR (("Thorax"[Mesh]) AND (("Diagnostic Imaging"[Mesh]) OR ("Radiography"[Mesh] OR "X-Rays"[Mesh])))) AND ((((HIV OR hiv OR human immunodeficiency virus OR human immunedeficiency virus OR hiv-1 OR hiv-2 OR hiv infections OR hiv positive OR HIV seropositivity OR AIDS OR acquired immunodeficiency syndrome OR acquired immunedeficiency syndrome)) OR ("HIV Seropositivity"[Mesh] OR "HIV-1"[Mesh] OR "HIV-2"[Mesh] OR "HIV Infections"[Mesh] OR "HIV"[Mesh])) OR ("Acquired Immunodeficiency Syndrome"[Mesh]))) AND ((((pneumonia OR bronchopneumonia OR broncho-pneumonia OR community-acquired pneumonia OR respiratory tract infection OR respiratory infection OR respiratory disease OR respiratory illness OR pulmonary disease OR pulmonary illness OR lung infection or severe acute respiratory infection or lower respiratory tract infection)) OR ("Pneumonia"[Mesh])) OR ("Respiratory Tract Diseases"[Mesh]))) AND (((Pneumocystis jirovecii OR pneumocystis OR pneumocystis pneumonia OR pneumocystis jirovecii OR pneumocystis jiroveci OR pneumocystis carinii OR pneumocystis carini OR pneumocystosis OR pcp OR pjp OR pcc)) OR ("Pneumonia, Pneumocystis"[Mesh] OR "Pneumocystis"[Mesh] OR "Pneumocystis carinii"[Mesh] OR "Pneumocystis Infections"[Mesh])) |
| --- | --- |
| #22 | Search: **(((("x ray" OR x-ray OR xray OR radiography OR radiograph OR film OR imaging OR image OR X-ray OR "X ray" OR Xray)) AND ((chest OR thorax OR pulmonary))) OR ((CXR OR cxr))) OR (("Thorax"[Mesh]) AND (("Diagnostic Imaging"[Mesh]) OR ("Radiography"[Mesh] OR "X-Rays"[Mesh])))** |
| #21 | Search: **("Thorax"[Mesh]) AND (("Diagnostic Imaging"[Mesh]) OR ("Radiography"[Mesh] OR "X-Rays"[Mesh]))** |
| #20 | Search: **("Diagnostic Imaging"[Mesh]) OR ("Radiography"[Mesh] OR "X-Rays"[Mesh])** |
| #19 | Search: **"Radiography"[Mesh] OR "X-Rays"[Mesh]** Sort by: **Most Recent** |
| #18 | Search: **"Diagnostic Imaging"[Mesh]** Sort by: **Most Recent** |
| #17 | Search: **"Thorax"[Mesh]** Sort by: **Most Recent** |
| #16 | Search: **((("x ray" OR x-ray OR xray OR radiography OR radiograph OR film OR imaging OR image OR X-ray OR "X ray" OR Xray)) AND ((chest OR thorax OR pulmonary))) OR ((CXR OR cxr))** |
| #15 | Search: **(CXR OR cxr)** |
| #14 | Search: **(("x ray" OR x-ray OR xray OR radiography OR radiograph OR film OR imaging OR image OR X-ray OR "X ray" OR Xray)) AND ((chest OR thorax OR pulmonary))** |
| #13 | Search: **(chest OR thorax OR pulmonary)** |
| #12 | Search: **("x ray" OR x-ray OR xray OR radiography OR radiograph OR film OR imaging OR image OR X-ray OR "X ray" OR Xray)** |
| #11 | Search: **(((HIV OR hiv OR human immunodeficiency virus OR human immunedeficiency virus OR hiv-1 OR hiv-2 OR hiv infections OR hiv positive OR HIV seropositivity OR AIDS OR acquired immunodeficiency syndrome OR acquired immunedeficiency syndrome)) OR ("HIV Seropositivity"[Mesh] OR "HIV-1"[Mesh] OR "HIV-2"[Mesh] OR "HIV Infections"[Mesh] OR "HIV"[Mesh])) OR ("Acquired Immunodeficiency Syndrome"[Mesh])** |
| #10 | Search: **"Acquired Immunodeficiency Syndrome"[Mesh]** Sort by: **Most Recent** |
| #9 | Search: **"HIV Seropositivity"[Mesh] OR "HIV-1"[Mesh] OR "HIV-2"[Mesh] OR "HIV Infections"[Mesh] OR "HIV"[Mesh]** Sort by: **Most Recent** |
| #8 | Search: **(HIV OR hiv OR human immunodeficiency virus OR human immunedeficiency virus OR hiv-1 OR hiv-2 OR hiv infections OR hiv positive OR HIV seropositivity OR AIDS OR acquired immunodeficiency syndrome OR acquired immunedeficiency syndrome)** |
| #7 | Search: **(((pneumonia OR bronchopneumonia OR broncho-pneumonia OR community-acquired pneumonia OR respiratory tract infection OR respiratory infection OR respiratory disease OR respiratory illness OR pulmonary disease OR pulmonary illness OR lung infection or severe acute respiratory infection or lower respiratory tract infection)) OR ("Pneumonia"[Mesh])) OR ("Respiratory Tract Diseases"[Mesh])** |
| #6 | Search: **"Respiratory Tract Diseases"[Mesh]** Sort by: **Most Recent** |
| #5 | Search: **"Pneumonia"[Mesh]** Sort by: **Most Recent** |
| #4 | Search: **(pneumonia OR bronchopneumonia OR broncho-pneumonia OR community-acquired pneumonia OR respiratory tract infection OR respiratory infection OR respiratory disease OR respiratory illness OR pulmonary disease OR pulmonary illness OR lung infection or severe acute respiratory infection or lower respiratory tract infection)** |
| #3 | Search: **((Pneumocystis jirovecii OR pneumocystis OR pneumocystis pneumonia OR pneumocystis jirovecii OR pneumocystis jiroveci OR pneumocystis carinii OR pneumocystis carini OR pneumocystosis OR pcp OR pjp OR pcc)) OR ("Pneumonia, Pneumocystis"[Mesh] OR "Pneumocystis"[Mesh] OR "Pneumocystis carinii"[Mesh] OR "Pneumocystis Infections"[Mesh])** |
| #2 | Search: **"Pneumonia, Pneumocystis"[Mesh] OR "Pneumocystis"[Mesh] OR "Pneumocystis carinii"[Mesh] OR "Pneumocystis Infections"[Mesh]** Sort by: **Most Recent** |
| #1 | Search: **(Pneumocystis jirovecii OR pneumocystis OR pneumocystis pneumonia OR pneumocystis jirovecii OR pneumocystis jiroveci OR pneumocystis carinii OR pneumocystis carini OR pneumocystosis OR pcp OR pjp OR pcc)** |

**Appendix 2. Study quality and Risk of Bias assessment tool**

This tool is based on the Newcastle-Ottawa scale^[55]^ and adapted for this review’s objectives, with descriptive selection and attrition bias assessment according to Cochrane Risk of Bias tool^[56]^.

1. **Study design and sampling frame**

a. Prospective/cross sectional clinical or laboratory studies (any type)

- Consecutive enrolment 2

- Unspecified/random enrolment 1

b. Autopsy studies

- Consecutive enrolment 2

- Unspecified/random enrolment 1

c. Retrospective reviews (including subgroup analysis) 1

d. Review/editorial 0

e. Case report 0

2. **Sample size calculation**

1. Sample size calculated and target reached 2
2. Sample size calculated, but target not reached or inputs unclear 1
3. No evidence of/unclear sample size target or calculation 1 – describe further in “selection bias”

3. **Selection of participants**

a. Clear inclusion criteria

- Primary objectives related to PCP clinical/radiological features or outcomes and clear reporting of inclusion and exclusion criteria 3

- Related to OIs/general morbidity/respiratory disease but not specific to PCP, or related to PCP but either inclusion or exclusion criteria are unclear 2

- Unrelated to PCP clinical/radiological features 1

b. Unclear inclusion/exclusion criteria 1

Assess representativeness of investigated participants separately (selection bias) below

4. **Outcome data reliability: diagnosis and case definitions**

1. Clear definitions of laboratory-confirmed diagnoses AND clear description of CXR assessment method with blinding to clinical/laboratory results of patients – 2
2. CXR categories and laboratory respiratory-diagnostic methods mentioned but not well described and/or CXR review not systematic or blinded 1
3. No description of diagnostic methods or CXR assessment methods 0

5. **Outcome data reliability and reporting: denominator (number of adults with HIV investigated for PCP ± other laboratory-confirmed respiratory diagnoses and have had CXR review)**

a. Raw data denominator 2

b. Calculated denominator 1

c. No/unclear denominator 0

6. **Outcome data reliability and reporting: numerator (number of adults with HIV and with PCP ± other laboratory-confirmed respiratory diagnoses with CXR feature of interest)**

a. Raw data numerator, all data presented 2

b. Calculated numerator data 1

c. No/unclear or significant (>50%) missing numerator data 0

7. **Assessment of bias (low, high or unclear risk)**

a. Attrition bias in cohort studies (loss to follow up, missing outcome data that is not accounted for)

- Amount, nature or handling of incomplete outcome data

b. Selection bias in cross sectional and cohort studies

- Representativeness of the cases/cohort (clear reasons for and rates of non-inclusion)

For these biases, and any other bias detected:

Low risk: study appears to be free of bias

High risk: there is at least one important risk of bias that could alter the CXR features reported

Unclear risk: there may be a risk of bias, but there is either (1) insufficient information to assess whether an important risk of bias exists or (2) insufficient rationale or evidence that an identified problem will introduce bias.

Quality scores: ≥ 12 = good; 10 - 11 = moderate; ≤ 9= poor quality

Score of 0 for any item: exclude from review.

**References**

1. Hansell DM, Bankier AA, MacMahon H, McLoud TC, Müller NL, Remy J. Fleischner Society: Glossary of terms for thoracic imaging. Radiology. 2008;246(3):697–722.

2. Dawson R, Masuka P, Edwards DJ, Bateman ED, Bekker LG, Wood R, et al. Chest radiograph reading and recording system: Evaluation for tuberculosis screening in patients with advanced HIV. Int J Tuberc Lung Dis. 2010;14(1):52–8.

3. Ryu JH, Olson EJ, Midthun DE, Swense SJ. Diagnostic approach to the patient with diffuse lung disease. Mayo Clin Proc. 2002;77(11):1221–7.

4. Christe A, Walti L, Charimo J, Rauch A, Furrer H, Meyer A, et al. Imaging patterns of Pneumocystis jirovecii pneumonia in HIV-positive and renal transplant patients - a multicentre study. Swiss Med Wkly. 2019 Oct;149(October):w20130.

5. Chave JP, Bille J, Glauser MP, Francioli P. Diagnosis of pulmonary infections in patients infected with the human immunodeficiency virus. Eur J Clin Microbiol Infect Dis. 1989;8(2):123–6.

6. Garay SM, Greene J. Prognostic indicators in the initial presentation of Pneumocystis carinii pneumonia. Chest [Internet]. 1989 Apr;95(4):769–72. Available from: http://dx.doi.org/10.1378/chest.95.4.769

7. Marggrander DT, Koc-Guenel S, Tekeli-Camci N, Martin S, Golbach R, Wolf T. Lung Ultrasound Effectively Detects HIV-Associated Interstitial Pulmonary Disease. Int J Infect Dis IJID Off Publ Int Soc Infect Dis Infect Dis. 2021 Oct;111:204–10.

8. Stover DE, White DA, Romano PA, Gellene RA, Robeson WA. Spectrum of pulmonary diseases associated with the acquired immune deficiency syndrome. Am J Med. 1985 Mar;78(3):429–37.

9. Batungwanayo J, Taelman H, Lucas S, Bogaerts J, Alard D, Kagame A, et al. Pulmonary disease associated with the human immunodeficiency virus in Kigali, Rwanda. A fiberoptic bronchoscopic study of 111 cases of undetermined etiology. Am J Respir Crit Care Med. 1994 Jun;149(6):1591–6.

10. Hargreaves N, Kadzakumanja O, Phiri S, Lee CH, Tang X, Salaniponi FM, et al. Pneumocystis carinii pneumonia in patients being registered for smear-negative pulmonary tuberculosis in Malawi. Trans R Soc Trop Med Hyg [Internet]. 2001;95(4):402–8. Available from: http://ovidsp.ovid.com/ovidweb.cgi?T=JS&PAGE=reference&D=emed8&NEWS=N&AN=33533623

11. Schlossbauer T, Schmidt GP, Bogner JR, Sing A, Reiser MF, Becker-Gaab C. Pulmonary radiological characteristics in patients with HIV infection at the time of highly active antiretroviral therapy (HAART). Eur J Med Res [Internet]. 2007;12(8):341–6. Available from: https://www.scopus.com/inward/record.uri?eid=2-s2.0-34548502452&partnerID=40&md5=15b82a452116eb979977410e00d980bc

12. Heron CW, Hine AL, Pozniak AL, Swinburn CR, Johnson NM. Radiographic features in patients with pulmonary manifestations of the acquired immune deficiency syndrome. Clin Radiol. 1985 Nov;36(6):583–8.

13. Minor O Le, Germani Y, Chartier L, Lan NH, Lan NTP, Duc NH, et al. Predictors of pneumocystosis or tuberculosis in HIV-infected asian patients with AFB smear-negative sputum pneumonia. J Acquir Immune Defic Syndr. 2008;48(5):620–7.

14. Malin AS, Gwanzura LK, Klein S, Robertson VJ, Musvaire P, Mason PR. Pneumocystis carinii pneumonia in Zimbabwe. Lancet (London, England) [Internet]. 1995;346(8985):1258–61. Available from: http://ovidsp.ovid.com/ovidweb.cgi?T=JS&PAGE=reference&D=med3&NEWS=N&AN=7475717

15. Mateyo KJ, Lakhi S, Guffey B, Chi B, Mweemba A, Andrews B. Pulmonary disease in HIV-infected Patients at the University Teaching Hospital, Lusaka, Zambia. Med J Zambia [Internet]. 2015;41(2):50–8. Available from: https://www.ajol.info/index.php/mjz/article/view/121204

16. Pozniak AL, Tung KT, Swinburn CR, Tovey S, Semple SJ, Johnson NM. Clinical and bronchoscopic diagnosis of suspected pneumonia related to AIDS. Br Med J (Clin Res Ed). 1986 Sep;293(6550):797–9.

17. Selwyn P, Pumerantz A, Durante A, Alcabes P, Gourevitch M, Boiselle P, et al. Clinical predictors of Pneumocystis carinii pneumonia, bacterial pneumonia and tuberculosis in HIV-infected patients. AIDS. 1998 May;12(8):885–93.

18. Suster B, Akerman M, Orenstein M, Wax MR. Pulmonary manifestations of AIDS: review of 106 episodes. Radiology. 1986 Oct;161(1):87–93.

19. Amin Z, Miller RF, Shaw PJ. Lobar or segmental consolidation on chest radiographs of patients with HIV infection. Clin Radiol. 1997 Jul;52(7):541–5.

20. Amorosa JK, Nahass RG, Nosher JL, Gocke DJ. Radiologic distinction of pyogenic pulmonary infection from Pneumocystis carinii pneumonia in AIDS patients. Radiology. 1990 Jun;175(3):721–4.

21. Ansari NA, Kombe AH, Kenyon TA, Hone NM, Tappero JW, Nyirenda ST, et al. Pathology and causes of death in a group of 128 predominantly HIV-positive patients in Botswana, 1997-1998. Int J Tuberc Lung Dis [Internet]. 2002;6(1):55–63. Available from: http://ovidsp.ovid.com/ovidweb.cgi?T=JS&PAGE=reference&D=med4&NEWS=N&AN=11931402

22. Estrada Chacón U, Bandera Tirado JF, Portela Ramirez D, Benavides García S. Alteraciones radiológicas en pacientes VIH con infección respiratoria aguda. Rev Cubana Med [Internet]. 2002;41(6). Available from: https://www.scopus.com/inward/record.uri?eid=2-s2.0-52649133414&partnerID=40&md5=62694508c054ea0c255b40bea5ef6da8

23. Kibiki G, Beckers P, Mulder B, Arens T, Mueller A, Boeree MJ, et al. Aetiology and presentation of HIV/AIDS-associated pulmonary infections in patients presenting for bronchoscopy at a referral hospital in northern Tanzania. East Afr Med J [Internet]. 2007;84(9):420–8. Available from: http://ovidsp.ovid.com/ovidweb.cgi?T=JS&PAGE=reference&D=emed11&NEWS=N&AN=350157399

24. Weinberg A, Duarte MI. Respiratory complications in Brazilian patients infected with human immunodeficiency virus. Rev Inst Med Trop Sao Paulo. 1993;35(2):129–39.

25. Baughman RP, Dohn MN, Shipley R, Buchsbaum JA, Frame PT. Increased pneumocystis carinii recovery from the upper lobes in pneumocystis pneumonia; The effect of aerosol pentamidine prophylaxis. Chest. 1993 Feb;103(2):426–32.

26. de la Paz Bermúdez T, González IGG, de Paz VCC, Ramírez DPP, Jiménez Pérez NAA, Sandubeti ECC, et al. Radiological findings of pneumocystis jirovecii pneumonia in Cuban deceased HIV/AIDS patients. Rev Cubana Med Trop [Internet]. 2020;72(2):1–13. Available from: https://www.scopus.com/inward/record.uri?eid=2-s2.0-85091720779&partnerID=40&md5=6195a496515b26c583dcfbff81e8ee91

27. Duflo B, Goyet F, Rozenbaum W, Rosenheim M, Datry A, Mayaud C, et al. Pneumocystosis among AIDS patients. About 21 cases. Med Mal Infect [Internet]. 1986;16(5):296–9. Available from: https://www.scopus.com/inward/record.uri?eid=2-s2.0-0022601246&doi=10.1016%2FS0399-077X%2886%2980236-0&partnerID=40&md5=c5c632625c2fe487d6848b9de8954019

28. Edelstein H, McCabe RE. Atypical presentations of Pneumocystis carinii pneumonia in patients receiving inhaled pentamidine prophylaxis. Chest. 1990 Dec;98(6):1366–9.

29. Engelberg LA, Lerner CW, Tapper ML. Clinical features of Pneumocystis pneumonia in the acquired immune deficiency syndrome. Am Rev Respir Dis. 1984 Oct;130(4):689–94.

30. Kaouech E, Kallel K, Anane S, Belhadj S, Abdellatif S, Mnif K, et al. [Pnemocystis jiroveci pneumonia: Comparison between conventional PCR and staining techniques]. Pathol Biol. 2009 Jul;57(5):373–7.

31. Leach R, Davidson A, Odoherty M, Nayagam M, Tang A, Bateman N. Non-invasive management of fever and breathlessness in HIV positive patients. Eur Respir J. 1991 Jan;4(1):19–25.

32. Mones JM, Saldana MJ, Oldham SA. Diagnosis of Pneumocystis carinii pneumonia. Roentgenographic-pathologic correlates based on fiberoptic bronchoscopy specimens from patients with the acquired immunodeficiency syndrome. Chest. 1986 Apr;89(4):522–6.

33. Peruzzi WT, Skoutelis A, Shapiro BA, Murphy RM, Currie DL, Cane RD, et al. Intensive care unit patients with acquired immunodeficiency syndrome and Pneumocystis carinii pneumonia: suggested predictors of hospital outcome. Crit Care Med. 1991 Jul;19(7):892–900.

34. Wang H, Li T, Wang A, Sheng R, Fan H, Liu Z, et al. A clinical analysis of 22 cases of Pneumocystis pneumonia in acquired immunodeficiency syndrome. Zhonghua Nei Ke Za Zhi [Internet]. 2005;44(9):652–5. Available from: https://www.scopus.com/inward/record.uri?eid=2-s2.0-33746085289&partnerID=40&md5=87000470b5e36d2d69f5a59f04514906

35. Wollschlager CM, Khan FA, Chitkara RK, Shivaram U. Pulmonary manifestations of the acquired immunodeficiency syndrome (AIDS). Chest. 1984 Feb;85(2):197–202.

36. Brenner M, Ognibene FP, Lack EE, Simmons JT, Suffredini AF, Lane HC, et al. Prognostic factors and life expectancy of patients with acquired immunodeficiency syndrome and Pneumocystis carinii pneumonia. Am Rev Respir Dis. 1987 Nov;136(5):1199–206.

37. Choi J, Lee K, Cho C, Han S, Choi S, Chin B, et al. Comparison of Clinical Characteristics of Pneumocystis Carinii Pneumonia between HIV Infected and Non-Infected Persons. Tuberc Respir Dis (Seoul). 2003;55(4):370–7.

38. DeLorenzo LJ, Huang CT, Maguire GP, Stone DJ. Roentgenographic patterns of Pneumocystis carinii pneumonia in 104 patients with AIDS. Chest. 1987 Mar;91(3):323–7.

39. Ewig S, Schafer H, Rockstroh JK, Pickenhain A, Luderitz B. Effect of long-term primary aerosolized pentamidine prophylaxis on breakthrough Pneumocystis carinii pneumonia. Eur Respir J. 1996 May;9(5):1006–12.

40. Griffiths MH, Miller RF, Semple SJ. Interstitial pneumonitis in patients infected with the human immunodeficiency virus. Thorax. 1995 Nov;50(11):1141–6.

41. Mane A, Gujar P, Chandra J, Lokhande R, Dhamgaye T, Ghorpade S, et al. Pneumocystis jirovecii infection and the associated dihydropteroate synthase (DHPS) and dihydrofolate reductase (DHFR) mutations in HIV-positive individuals from Pune, India. Mycopathologia. 2015 Feb;179(1–2):141–5.

42. Silva RM da, Bazzo ML, Borges AA. Induced sputum versus bronchoalveolar lavage in the diagnosis of pneumocystis jiroveci pneumonia in human immunodeficiency virus-positive patients. Braz J Infect Dis. 2007 Dec;11(6):549–53.

43. McLeod DT, Neill P, Gwanzura L, Latif AS, Emmanuel JC, Nkanza N, et al. Pneumocystis carinii pneumonia in patients with AIDS in Central Africa. Respir Med. 1990 May;84(3):225–8.

44. Miller RF, Millar AB, Weller I V, Semple SJ. Empirical treatment without bronchoscopy for Pneumocystis carinii pneumonia in the acquired immunodeficiency syndrome. Thorax. 1989 Jul;44(7):559–64.

45. Opravil M, Marincek B, Fuchs W, Weber R, Speich R, Battegay M, et al. Shortcomings of chest radiography in detecting Pneumocystis carinii pneumonia. J Acquir Immune Defic Syndr. 1994 Jan;7(1):39–45.

46. Rozaliyani A, Wiyono WH, Nawas MA, Sjam R, Adawiyah R, Tugiran M, et al. Laboratory findings and clinical characteristics of Pneumocystis pneumonia and tuberculosis infection among HIV-infected patients with pulmonary infiltrates in Jakarta, Indonesia. Trop Biomed. 2020 Dec;37(4):1117–23.

47. Udwadia ZF, Doshi A V., Bhaduri AS. Pneumocystis Carinii pneumonia in HIV infected patients from Mumbai. J Assoc Physicians India. 2005;53(May):437–40.

48. Cohen BA, Pomeranz S, Rabinowitz JG, Rosen MJ, Train JS, Norton KI, et al. Pulmonary complications of AIDS: Radiologic features. Am J Roentgenol [Internet]. 1984;143(1):115–22. Available from: https://www.scopus.com/inward/record.uri?eid=2-s2.0-0021236045&doi=10.2214%2Fajr.143.1.115&partnerID=40&md5=19189cf1bd8428c4868885b8bdf17dde

49. Diero L, Stiffler T, Einterz RM, Tierney WM. Can data from an electronic medical record identify which patients with pneumonia have Pneumocystis carinii Infection [Internet]. Vol. 73, International Journal of Medical Informatics. 2004. p. 743–50. Available from: http://search.ebscohost.com/login.aspx?direct=true&AuthType=cookie,ip,shib&db=awn&AN=15491925&site=ehost-live

50. Rosen MJ, Tow TW, Teirstein AS, Chuang MT, Marchevsky A, Bottone EJ. Diagnosis of pulmonary complications of the acquired immune deficiency syndrome. Thorax. 1985 Aug;40(8):571–5.

51. Siika AM, Chakaya JM, Revathi G, Mohamed SS, Bhatt KM. Bronchoscopic study on aetiology of chronic cough in HIV-infected adults with negative sputum smears for Mycobacterium tuberculosis at Kenyatta National Hospital, Nairobi. East Afr Med J [Internet]. 2006;83(6):295–305. Available from: http://ovidsp.ovid.com/ovidweb.cgi?T=JS&PAGE=reference&D=emed10&NEWS=N&AN=44358311

52. Huang L, Stansell J, Osmond D, Turner J, Shafer KP, Fulkerson W, et al. Performance of an algorithm to detect Pneumocystis carinii pneumonia in symptomatic HIV-infected persons. Pulmonary Complications of HIV Infection Study Group. Chest. 1999 Apr;115(4):1025–32.

53. Logan P, Primack S, Staples C, Miller R, Muller N. Acute lung disease in the immunocompromised host. Diagnostic accuracy of the chest radiograph. Chest. 1995 Nov;108(5):1283–7.

54. Orlovic D, Kularatne R, Ferraz V, Smego RAJ. Dual pulmonary infection with Mycobacterium tuberculosis and Pneumocystis carinii in patients infected with human immunodeficiency virus. Clin Infect Dis [Internet]. 2001;32(2):289–94. Available from: http://ovidsp.ovid.com/ovidweb.cgi?T=JS&PAGE=reference&D=med4&NEWS=N&AN=11170920

55. Wells G, Shea B, O’Connell D, Peterson J, Welch V, Losos M, et al. The Newcastle-Ottawa Scale (NOS) for assessing the quality of nonrandomised studies in meta-analyses [Internet]. Available from: https://www.ohri.ca/programs/clinical_epidemiology/oxford.asp

56. The Cochrane Collaboration. Editors: Higgins J, Green S. Cochrane Handbook for Systematic Reviews of Interventions [Internet]. 2011. Chapter 8: Assessing risk of bias in included stud. Available from: https://handbook-5-1.cochrane.org/front_page.htm
